# Anomanolide C suppresses tumor progression and metastasis by ubiquitinating GPX4-driven autophagy-dependent ferroptosis in triple negative breast cancer

**DOI:** 10.7150/ijbs.82120

**Published:** 2023-05-08

**Authors:** Yan-Mei Chen, Wei Xu, Yang Liu, Jia-Hui Zhang, Yuan-Yuan Yang, Zhi-wen Wang, De-Juan Sun, Hua Li, Bo Liu, Li-Xia Chen

**Affiliations:** 1 Wuya College of Innovation, School of Traditional Chinese Materia Medica, Key Laboratory of Structure-Based Drug Design & Discovery, Ministry of Education, Shenyang Pharmaceutical University, Shenyang, 110000, China.; 2 State Key Laboratory of Biotherapy and Cancer Center, West China Hospital, Sichuan University, Chengdu, 610041, China.; 3 Institute of Structural Pharmacology & TCM Chemical Biology, College of Pharmacy, Fujian University of Traditional Chinese Medicine, Fuzhou 350122, China.

**Keywords:** Anomanolide C, Triple negative breast cancer, Autophagy, Ferroptosis, GPX4, Ubiquitination

## Abstract

Anomanolide C (AC), a natural withanolide isolated from *Tubocapsicum anomalum*, has been reported to have exhibits remarkable anti-tumour activities in several types of human cancers, particularly triple-negative breast cancer (TNBC). However, its intricate mechanisms still remain need to be clarified. Here, we evaluated whether AC could inhibit cell proliferation and the role of AC in ferroptosis induction and autophagy activation. Subsequently, the anti-migration potential of AC was found via autophagy-dependent ferroptosis*.* Additionally, we found that AC reduced the expression of GPX4 by ubiquitination and inhibited TNBC proliferation and metastasis *in vitro* and *in vivo.* Moreover, we demonstrated that AC induced autophagy-dependent ferroptosis, and led to Fe^2+^ accumulation *via* ubiquitinating GPX4. Moreover, AC was shown to induce autophagy-dependent ferroptosis as well as to inhibit TNBC proliferation and migration *via* GPX4 ubiquitination. Together, these results demonstrated that AC inhibited the progression and metastasis of TNBC by inducing autophagy-dependent ferroptosis *via* ubiquitinating GPX4, which might shed light on exploiting AC as a new drug candidate for the future TNBC therapy.

## Introduction

Breast cancer (BC) is the most common cancer in females on a global scale, which is characterized by high morbidity and mortality rates 1-4. The onset age of breast cancer tends to be younger in China, with the fastest increase in mortality in the past 10 years 5-8. One of the heterogeneous BC subtypes is triple-negative breast cancer (TNBC), which lacks three molecular targets: estrogen receptor (ER), human epidermal growth factor receptor-2 (HER-2), and progesterone receptor (PR) 9. Further, this type is associated with poor clinical outcomes. So far, clinical strategies have been developed, including chemotherapy and targeted therapy. However, multi-drug resistance and metastasis of tumor cells have reduced the scope of clinical applications of chemotherapy drugs to some extent 10-13. Therefore, it is necessary to explore new small-molecule compounds for TNBC therapy.

Recently, numerous reports have demonstrated that the pathogenesis of breast cancer is related to many vital events, such as autophagy, ferroptosis, and other subroutines of regulated cell death (RCD) 14-17. Autophagy Cell death-dependent cell death is highly complicated, and has an intricate relationships with different forms of RCD, such as ferroptosis 18, 19. Ferroptosis has been reported as an oxidative stress and iron-dependent form of RCD 20. Accumulating evidence has revealed that ferroptosis is autophagy-dependent in many types of human cancers 21. Further, ferroptosis is characterized by the accumulation of ferrous ion (Fe^2+^) and lipid peroxidation 22-24. Iron-mediated oxidative stress is a key mechanism during ferroptosis 25, 26. Ferroptosis mediates ferritin degradation, and ferritin usually contains the ferritin heavy chain (FTH) and ferritin light chain (FTL) 27, 28. FTH catalyzes the oxidation of Fe^2+^ ion, which is the principal step in iron storage. Thus, the induction of autophagy-dependent ferroptosis is a promising strategy for suppressing cancer cells progression 29, 30.

Withanolides, a kind of highly-oxidized C28 ergosterol steroids, mainly exist in plants of Solanaceae family and have been attracting a rising interest because of their biological characteristics, especially anti-cancer activity 31. Previous studies have reported that the anti-tumor effect of withanolides depends on both α, β-unsaturated ketone in ring A and 5β, 6β-epoxy group in ring B 32, 33. *Tubocapsicum anomalum*, belonging to the family of Solanaceae, is widespread in southern China and has been reported to be the source of withanolides 34, 35, 36. Anomanolide C (AC) is a major active constituent of *Tubocapsicum anomalum,* showing potent anti-cancer activity 36-39. However, its potential targets and relevant mechanisms have greatly hindered its therapuetic applications as a candidate anticancer drug.

Thus, in this study, we demonstrated whether AC can inhibited TNBC progression and metastasis by inducing autophagy-dependent ferroptosis *via* ubiquitinating GPX4, which provide a clue for developing AC as a candidate compound from herbal medicine to treat TNBC.

## Materials and methods

### Cell culture and reagents

All cells were purchased from American Type Culture Collection (Manassas, VA, USA). MCF-10A, HepG3B, MDA-MB-436, and BT549 cell lines were cultured in RPMI medium and the MDA-MB-231, SW480, MKN74, HCT116, HepG2, MDA-MB-231-luc, GSC7901, MCF-7, MDA-MB-468, Hela and A549 cell lines were cultured in DMEM with 1% penicillin-streptomycin (Life Technologies) and 10% fetal bovine serum (FBS) in 5% CO_2_ at 37°C. Cell lines were grown seeded in plates or cell culture dishes or plates to 80-90% confluence and all the tests were performed using cell at the logarithmic phase.

The protein of GPX4 was purchased from OriGene (Jiangsu, China). DAPI (D9542) and Fluorescein sodium salt (F6377) were bought from Sigma-Aldrich (St. Louis, MO, USA). Hoechst33258 (C0021) was acquired from Solarbio (Beijing, China). FerroOrange (F374) was obtained from Dojindo (Goryo Chemical Inc., Hokkaido, Japan). JC-1 Apoptosis Detection Kit (KGA601-KGA604) was purchased from KeyGEN BioTECH (Jiangsu, China). Mito SOXTM Red mitochondrial superoxide (M36008) and Reactive Oxygen Species (88-5930-74) were acquired from Thermo Fisher Scientific (USA). 3-MA (HY-19312), BafA1 (CSN10374-001), Fer-1 (HY-100579), MG132 (HY-13259), and Necrostatin-1 (Nec-1, HY-15760) were acquired from MedChemExpress (Shanghai, China). Ammonium tetrathiomolybdate (VI, CSN96630-005), Disulfiram (CSN10460-001) were purchased from Csnpharma (Shanghai, China). The following antibodies were applied to this study: β-actin (66009-1-Ig, Proteintech), Atg5 (9980s, CST), Atg7 (8558s, CST), ACSL4 (ET7111-43, HuaBio), Beclin1 (3495, CST), E-cadherin (M1405-3, HuaBio), FTH1 (R1601-9, HuaBio), GPX4 (ab252833, Abcam), GPX4 (ER1803-15, HuaBio), Ki67 (9449, CST), LC3B (51520, Abcam), MMP-3 (14351s, CST), MMP-2 (87809, CST), N-Cadherin (ET1607-37, HuaBio), SQSTM1/p62 (8025, CST), Vimentin (92547, Abcam), SLC7A11 (HA600098, HuaBio).

### Cell viability assay

The cell lines were distributed in 96-well plates (1 × 10^4^ cells/well). After 24 h of incubation at 37 °C, different concentrations of Anomanolide C dealt with cells for 24 h. CCK8 test was used to measured cell viability.

### GFP/mRFP - LC3 transfection

The cells lines were distributed on slide in 24-well plates (2 × 10^4^ cells/well). After 24 h of incubation, GFP/mRFP-LC3 (HB-AP2100001, HANBIO, China) was transfected into cells for 6 h, Then, cells were treated with AC for was added to treated 24 h followed by changing the culture medium 48 h. After transfection transfected 48 h, The cells were examined and tested further using a fluorescent microscope.

### Immunofluorescence (IF) analysis

Cells, from the 24-well plates, were transferred on to the glass slide and fixed for 30 minutes in PBS containing 4% paraformaldehyde. The glass slide was subsequently washed with PBS thrice (each time for 5 minutes) and hatched with 5% goat serum (G9023, Sigma-Aldrich) and 0.2%Triton X-100 (9002-93-1, Sigma-Aldrich) for 15 minutes. Cells, fixed on the slides, were hatched with indicated primar antibody at 4 °C overnight, use TBS/0.1% Tween-20 to wash the membrane thrice, then treated with secondary antibody for 1 h (Room temperature) (TRITC, ab6718; FITC, ab6717). Confocal laser scanning (Zeiss) was performed after staining the nuclei with DAPI for 5 minutes, and the images were examined.

### Immunohistochemistry (IHC) analysis

Sections of MDA-MB-231-luc mouse tumour and lungs were microwaved after immersion in citrate buffer in pH 6.0 or EDTA antigenic retrieval buffer in pH 8.0. These sections were hatched with antibodies (diluted at a 1:400 ratio) against MMP2, Ki67, GPX4, LC3B and E-cadherin for 1 h. Common mouse/rabbit IgG was considered as the negative control. and the slides were developed with diaminobenzidine solution after receiving a 30-min treatment with horseradish peroxidase (HRP)-conjugated secondary antibodies. Meyer's haematoxylin was used as the counterstain.

### Western blotting (WB) analysis

All cells, animal tumours, and lung tissues were suspended in the lysis buffer, incubated for 30 minutes at 4 °C, and centrifugated at 12,000 rpm for 10 minutes. The protein content of the supernatant was assessed using the Bio-Rad DC protein assay (Bio-Rad Laboratories, Hercules, CA, USA). Total proteins were separated using SDS-PAGE and transferred on to PVDF membrances. The membrances were first incubated with 5% skimmed milk by protein-specific primary antibodis, and finally with HRP-conjugated secondary antibodies. The membrances were subsequently incubated with the HRP substrate for visualisation using enhanced chemilumnescence (ECL). Densitometric analysis of proteins bands was conducted using the ImageJ software.

### Colony formation assay

Five hundred cells were transferred to 12-well plates and treated with AC or vehicle control to evaluate the proliferation potential of the cell lines in presence and absence of AC. After 4 weeks of incubation, cells were blended with methanol and stained using crystal violet. Then the colonies were subsequently tallied. And the date were represented as the average (and range) of three separate experiments performed in identical wells.

### Flow cytometric analysis of detecting ROS, mitochondrial ROS, Mitochondrial membrane potential, and free Fe^2+^ ion

Flow cytometry was applied to detect ROS, mitochondrial ROS (mito-ROS), mitochondrial membrane potential (MMP), and free ferrous iron. Briefly, AC-treated MDA-MB-231 and BT549 cells were centrifuged and washed thrice with PBS. DCFH-DA, Mito SOXTM red mitochondrial superoxide, JC-1, or FerroOrange were induced, and intracellular ROS were stained for 30 minutes at 37 °C. A FACS calibur instrument was used to collect the stained MDA-MB-231 and BT549 cells for analysis (Becton Dickinson, Mountain View, CA, USA).

### Scratch assay

In 6-well plates, the cells were grown seeded and scratched with sterile spear tips. Following a PBS wash, the cell lines were cultured in a regular medium or a medium containing AC. Under a microscope images were collected after a 24-hour incubation period.

### Transwell migration assay

After treatment with AC, the cells were collected and transferred on to the 8 µm pore size transwell filters (Millipore). Serum-free DMEM and DMEM with 10% FBS were respectively added to the bottom and top chambers, respectively. After 24 h of incubation, after fixation 4% PFA, the cells on the filters's top side were cleaned using cotton swabs, and the cells on the bottom side were stained with 0.1% crystal violet. The inverted microscope was used to capture images.

### Transfection

GeneChem produced si-Control, overexpression of GPX4 and si-ATG5 using synthetic methods (Shanghai, China). As the manufacturer's recommended protocol, RNA was transfected using Lipofectamine 3000 reagent (Thermo Fisher Scientific) for 48 h.

### Nude mice tumor models

Subcutaneous xenograft model: MDA-MB-231-luc cells (2 × 10^7^) or BT549 cells (2 × 10^6^) were subcutaneously injected into 30 female naked mice (BALB/c nude, 6-8 weeks, 18-20 g). Appoximatedly five days later when the grew a volume of 100 mm^3^ (V = L W^2^/2), three groups of mice were formed (normal group, normal; AC group, 25 mg/kg/day; AC group, 50 mg/kg/day). The weight of the mice was recorded daily during treatment, and data on MDA-MB-231-luc indueced nude mice in vivo imaging were recorded every week till the completion of the study. Besieds, tumour size of both two cells induced nude mice was measured with vernier caliper every three days. The heart, liver, spleen, kidney, lung and tumor tissues were gathered, weighted, photographed, and then instantly fixed in formalin or frozen in liquid nitrogen for later experimentation.

Intravenous xenograft model: MDA-MB-231-luc cells (2 × 10^7^) or BT549 cells (2 × 10^6^) were injected into 30 female nude mice (BALB/c nude, 6-8 weeks, 18-20g) intravenously. After five days of acclimatisation, three groups of nude mice were created: a normal group, an AC group receiving 25 mg/kg/day, and an AC group receiving 50 mg/kg/day. Date on naked mouse in vivo imaging and mouse weight were recorded weekly during treatment till the completion of the study. Lung tissues were photographed and immediately fixed with liquid nitrogen or formalin for subsequent experiments.

### 3D spheroids

3D spheroids model was carried out according to previous study 40, 41. Firstly, a total of 1 × 10^3^ tumor cells were inoculated in each well of U-bottom Ultra-Low Adherence 96-well plates (Dojindo, MS-9096UZ). Single spherical cells were formed in plates at 37 °C, 5% CO_2_, and 95% humidity for incubation of 72 h. Spheroids were then treated with AC in a fresh medium for the indicated time.

### CETSA assay

CETSA test was administered in accordance with a prior study 42, 43. AC (1 μM) was applied to MDA-MB-231 cells acting six hours at room temperature. Next, cells were hatched at the specified temperature for 3 times, each time for 5 minutes, Samples were frozen in liquid nitrogen and freeze-thaw cycle was liquid nitrogen repeatedly. Finally, the supernatant from a centrifuged at 12,000 rpm for 10 minutes is used for western blotting.

### Co-immunoprecipitation (Co-IP) assay

MDA-MB-231 and BT549 cells were cleaved on ice with RIPA lysates for 30 min. After 4,000 rpm centrifugation at 4 °C for 30 min, the supernatant was collected. A paucity of lysate was taken for immunoblotting analysis, and the remaining lysate was added with 1μg of corresponding antibody to the cell lysate, and incubated overnight with slow shaking at 4 °C. Then, 10 μL protein A agarose beads were washed three times with appropriate lysis buffer and centrifuged at 3,000 rpm each time for 3 min. The pretreated 10 μL protein A agarose beads were added to the cell lysate incubated with the antibody overnight and slowly shaken at 4 °C for 2-4 h to conjugate the antibody to the protein A agarose beads. After the immunoprecipitation reaction, agar-agar beads were centrifuged to the bottom of the tube at 3,000 rpm at 4 °C for 3 min. The supernatant was carefully sucked off and the agarose beads were washed with 1mL lysis buffer for 3-4 times. Finally, 15 μL of 2 × SDS loading buffer was added and boiled for 5 minutes. In addition, the samples were washed with PBS and observed with ECL detection system.

### Molecular docking and molecular dynamics (MD) simulations

The initial three dimensional geometric coordinates of the X-ray crystal structure of GPX4 (PDB code: 2OBI) was downloaded from the Protein Databank (https://www.rcsb.org/), and molecular docking was employed to estimate the interaction between AC and GPX4 pockets in screening by CDOCKER protocol 44. Besides, molecular dynamics (MD) simulations were performed according to our previous reports 45.

### Surface Plasmon Resonance (SPR)

The running buffer HBS-EP (10mM HEPES (pH 7.4), 150mM NaCl, 3mM EDTA and 0.5% (v/v) surfactant P20, 5% DMSO) was used to balance Biacore X200 instrument (GE Healthcare) at 25°C. Then, the GPX4 protein is first coupled to the surface of the CM5 chip biosensor in the environment of 10mM sodium acetate (pH 4.5). Meanwhile, the solution containing AC compounds is injected and flows through the biosensor surface at a flow rate of 20 μL/min for 120 seconds, and then the buffer solution is injected and flows through the biosensor surface for dissociation after 120 seconds. The combination of biomolecules causes the increase of the surface quality of the biosensor, leading to the change of the refractive index. By monitoring the angle change of SPR, the dissociation constant *K_D_* value of the analyte can be automatically obtained by using the Biacore T200 evaluation software version 1.0 (GE Healthcare). Changes in the reaction between biomolecules were observed.

### Quantitative real time PCR (qRT-PCR) assay

The total RNA was extracted by using Trizol reagent (TransGen Biotech, China) from MDA-MB-231 cells. Meanwhile, RNA quantity and purity were determined by using a NanoDrop 2000 (Thermo Scientific), and transcribed to cDNA using reverse transcription reagents (Fermentas China Co. Ltd., China). Relative mRNA expression was detected by RT-qPCR using 7500 RT-PCR System (Applied Biosystems, Life Technologies). An SYBR PrimeScript RT-PCR Kit (Cowin Biotech) was used according to the manufacturer's instructions. β-actin was used as loading controls. The primer sequences (Tsingke Biotechnology Co., Ltd., China) were listed in Supplementary file 1: Table S1.

### Statistical analysis

At least three separate experiments supported the data and findings. The data, shown as the means and standard error of means were analysed using GraphPad Prism 8.0 software. For statistical comparisons between two groups and among several groups, The Student's t-test and one-way analysis of variance was employed, respectively. Statistical significance was set at P < 0.05.

## Results

### Anomanolide C suppresses the proliferation of human TNBC cells *in vitro* and *in vivo*

**Figure 1:**
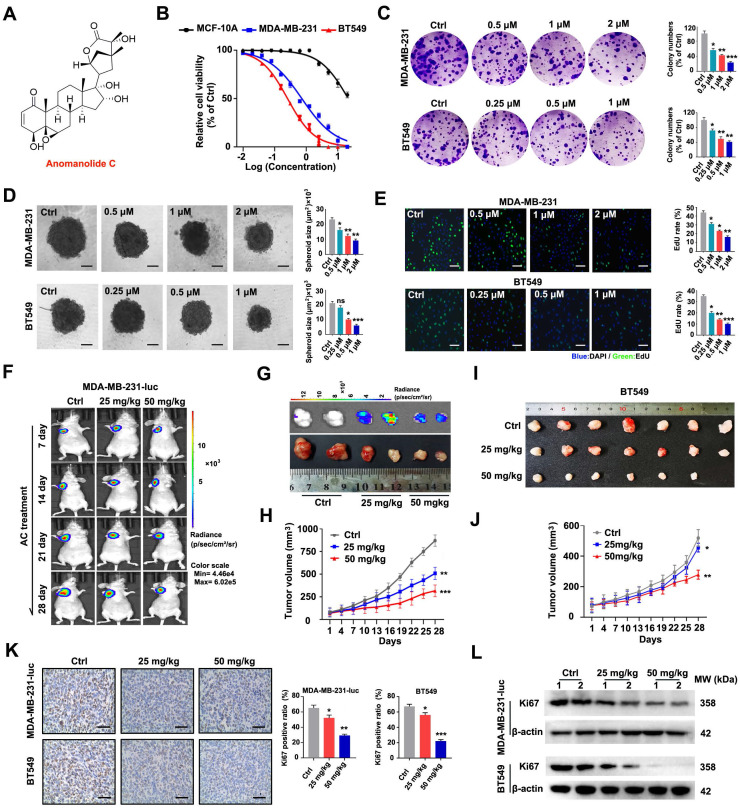
Anomanolide C prevents TNBC cell growth *in vivo* and *in vitro*. (A) The chemical structure of AC. (B) The IC_50_ values of AC in MCF-10A, BT549, MDA-MB-231 cells at different concentration. the IC_50_ values were calculated by Prism 8.0. (C) The colony formation of BT549 and MDA-MB-231 cells were tested in the presence and absence of AC (MDA-MB-231: 0.5, 1 and 2 μM; BT549: 0.25, 0.5, and 1 μM). Quantification of colonies and representative images are displayed. (D) MDA-MB-231 and BT549 3D spheroids with or without AC treatment were studied. Representative images and quantification of the 3D spheroids are presented. (E) EdU-DNA synthesis assay of BT549 and MDA-MB-231 cells were treated with different concentrations of AC for 24 h. Representative images and quantification of EdU-DNA synthesis are presented. (F) Fluorescence imaging of naked mice from different groups at 2 h post injection with or without AC (25 and 50 mg/kg). (G) Ex vivo fluorescence distribution imaging of tumor performed 2 h after the intraperitoneal injection of fluorescein potassium salt. Scale bar, 0.5 cm. (H) Quantitative analysis of the MDA-MB-231-luc cells tumor volume. (I) The photographs of tumors derived from naked mice in representative BT549 tumors *in vitro*. (J) Quantitative analysis of the BT549 cells tumor volume. (K) Immunohistochemical results of Ki67 expression in representative BT549 and MDA-MB-231-luc cells tumor sections of vehicle and AC (25 and 50 mg/kg) treated naked mice. Ki67 quantitation and representative images are shown. (L) Western blotting results of Ki67 expression in representative MDA-MB-231-luc and BT549 cells tumor sections of vehicle and AC (25 and 50 mg/kg) treated naked mice. Data are presented as mean ± SEM. Date are from at least three separate experiments. ns, not significant, *, *P* < 0.05, **, *P* < 0.01, ***, *P* < 0.001. Statistical significance was determined relative to the respective control groups.

To confirm the potential anti-cancer action of AC (Fig. 1A), cell counting kit-8 (CCK8) experiments were performed to measure the proliferation of several tumour cell lines after AC treatment. The survival ability of human cancer cells substantially decreased in a dose-dependent manner when treated with AC (Fig. S1A). Notably, TNBC cell line, MDA-MB-231 and BT549 were more susceptible to AC when treated for 24 h (IC_50_ values were 1.02 μM (MDA-MB-231) and 0.53 μM (BT549), respectively), compared with the human normal mammary epithelial cell, MCF-10A (Fig. 1B). Proliferation of BT549 and MDA-MB-231 cells was dramatically inhibited by AC treatment, as demonstrated by reducing colony formation (Fig. 1C). The proliferation of 3D spheroids also decreased following the AC treatment in BT549 and MDA-MB-231 cell cultures (Fig. 1D). We then conducted an EdU-DNA synthesis assay, and the synthesis of EdU-DNA verified that AC decreased the proliferative capacity of MDA-MB-231 and BT549 cells after AC treatment (Fig. 1E). These results show that AC is a potential *in vitro* anti-tumour agent, especially in MDA-MB-231 and BT549 cells.

To confirm the anti-tumour activity of AC *in vivo*, a mouse xenogenic in situ tumour model was established by subcutaneously inoculating MDA-MB-23-luc and BT549 cells into naked mice. Base on tumor imaging, tumor weight, and volume measurements, we discovered that xenogenic in situ tumor in mice treated with AC grew slower than that of the saline-treated tumors (Fig. 1F-J, S1B, C). When Ki67 was regarded as a biomarker to evaluate the proliferation of human cancer cells, we found that Ki67 staining was found to be consistently weaker after AC treatment than the control group (Fig. 1K). The immunoblotting assay further confirmed this result (Fig. 1L, S1D, E). There were no abnormalities in eating and behavioral activity of the nude mice that treated with or without AC (25 mg/kg, 50 mg/kg) throughout the whole experiment. The body weights were also increased in AC-treated groups, but the growth rate was slower, compared with the control groups (Fig. S1F, G). Furthermore, the major tissues (the heart, liver, spleen, lung, and kidney organs) were stained with H&E staining, demonstrating that AC did not have any obvious toxicity in the mice at the two doses (25 mg/kg, 50 mg/kg) of AC (Fig. S1H, I). These findings suggested that AC suppressed tumor growth *in vivo*. Collectively, these results show that AC can effectively prevent proliferation of BT549 and MDA-MB-231 cells.

### Anomanolide C inhibits metastasis of human TNBC cells *in vitro* and *in vivo*

**Figure 2:**
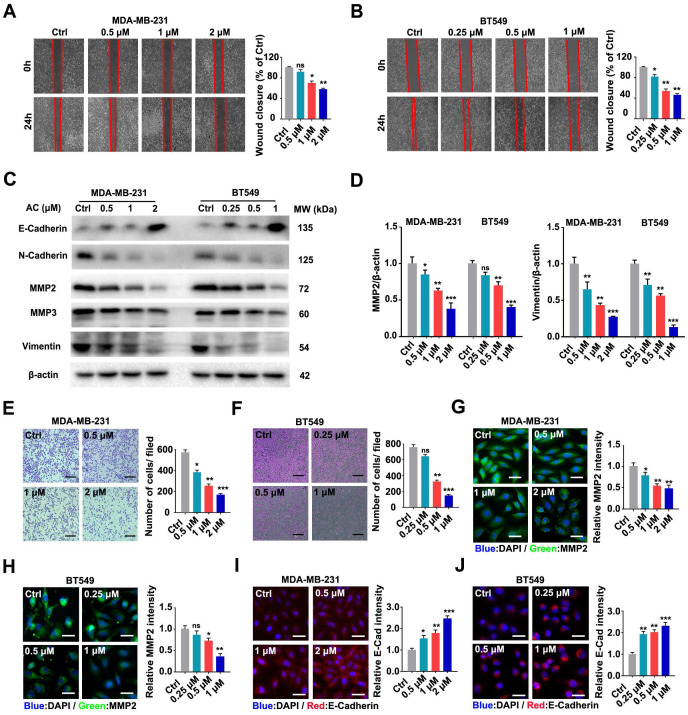
Anomanolide C inhibits the migration of human TNBC cells *in vitro*. (A) MDA-MB-231 cells were treated with or without AC (0.5, 1, and 2 μM) for 24 h. The weight movement capacity of the cells was determine using a scratch assay. The degree of cell migratory is represented by the wound closure ratio. Representative numbers and images are shown. Scale bar, 100 μm. (B) AC (0.25, 0.5, and 1 μM) was either added to or removed from BT549 cells for 24 h, and a scratch assay was used to measure the migration capabilities of the cells. The extent of a cell's ability to migrate is indicated by the wound-closure ratio. Representative images and facts are presented. Scale bar, 100 μm. (C) Immunoblotting analysis of MMP2, MMP3, Vimentin, N-Cadherin, and E-Cadherin expression in cells treated with AC for 24 h. beta-actin was evaluated as a loading control. (D) Quantified MMP2 and Vimentin levels are shown. (E-F) MDA-MB-231 and BT549 cells were dealt with or without AC for 24 h. The ability of the cells to migrate was evaluated using a transwell test. The degree of cell migration is represented by the wound ratio. Representative numbers and images are shown. Scale bar, 100 μm. (G-H) Immunofluorescence analysis of MMP2 levels in cells treated with or without AC for 24 h. Immunofluorescence images and quantification are shown. Scale bar, 20 μm. (I-J) Immunofluorescence analysis of E-cadherin levels in MDA-MB-231 and BT549 cells dealt with or without AC for 24 h. Immunofluorescence analysis was performed in a quantitative manner. Scale bar, 20 μm. Data are presented as the mean ± SEM. These results are consistent with those of at least three different experiments. ns, not significant, *, *P* < 0.05, **, *P* < 0.01, ***, *P* < 0.001. Statistical significance was determined to the respective control groups.

The prognosis of patients with breast cancer is poor because of frequent metastases, which occurs mostly in the lungs 46, 47. Hence, we further investigated the impact of AC on the scratch migration of BT549 and MDA-MB-231 cells and found that the TNBC cells wound closure rate decreased after the AC treatment (Fig. 2A, B). Immunoblotting analysis revealed that E-Cadherin was up-regulated, while MMP2, N-Cadherin, MMP3, and vimentin were down-regulated after AC treatment in TNBC cells (Fig. 2C, D, S2A-C). Moreover, transwell assays confirmed that AC treatment ultimately curbed the migration of BT549 and MDA-MB-231 cells (Fig. 2E, F). Furthermore, immunofluorescence analysis of cells treated with AC directly showed that increased E-cadherin and decreased MMP2 in the both cells (Fig. 2G-J). These results demonstrated that AC suppressed the migration of BT549 and MDA-MB-231 cells *in vitro*.

**Figure 3:**
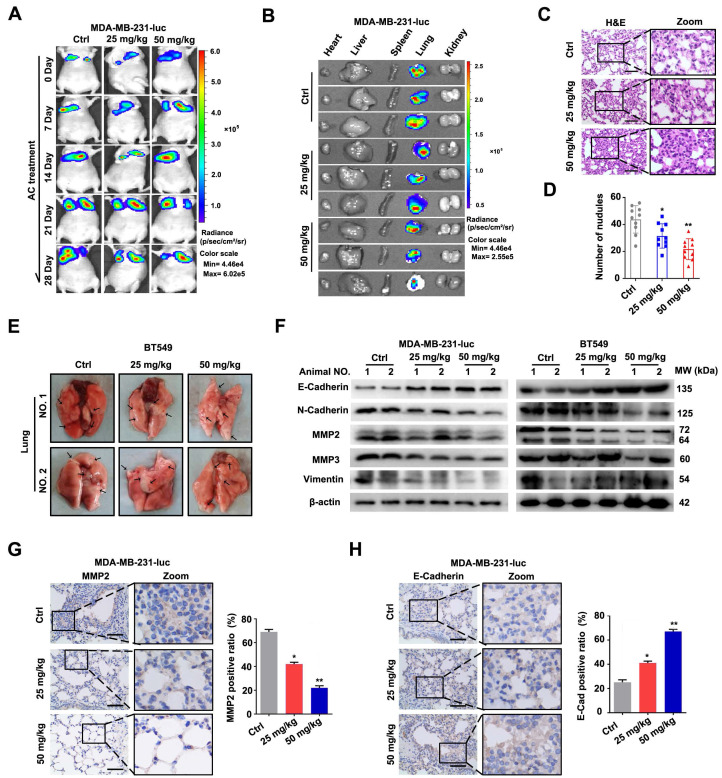
Anomanolide C inhibits the metastasis of human TNBC cells *in vivo*. (A) After a 2-hour intraperitoneal injection of fluorescein potassium salt, Mice from several groups treated with or without AC (25, 50 mg/kg) were fluorescently imaging. (B) After a 2-hour intraperitoneal injection of fluorescein potassium salt, the main organs were imaged using ex vivo fluorescence distribution. (C) Representative H&E staining of the lung slices of MDA-MB-231-luc induced nake mice-treated with or without AC (25 and 50 mg/kg). Scale bar, 40 μm. (D) The total number of metastatic nodules in the lungs of bare mice was used to measure the metastasis of MDA-MB-231 cells. (E) Representative images of lung metastasis from nude mice treated with or without AC, the BT549 cells injected into the vein of mice. The arrow represents pulmonary metastatic nodule. (F) Western blot analysis of MMP2, MMP3, vimentin, N-cadherin and E-cadherin expression in lung tissues of nude mice treated with and without AC (25 and 50 mg/kg). beta-actin was evaluated as a loading control. (G) Expression of MMP2 in representative lung sections of MDA-MB-231 treated nake mice from control or AC (25 and 50 mg/kg) groups. Quantified analysis and illustrative images are shown. Scale bar, 40 μm. (H) Expression of E-Cadherin in representative lung sections of MDA-MB-231 induced the nude mouse of control or AC (25 and 50 mg/kg)-treated groups. Quantified analysis and illustrative images are shown. Scale bar, 40 μm. Data are presented as the mean ± SEM. These results are consistent with those of at least three different experiments. ns, not significant, *, *P* < 0.05, **, *P* < 0.01, ***, *P* < 0.001. Statistical significance was determined relative to the respective control groups.

To further indicated the activity of AC *in vivo*, MDA-MB-231-luc cells were injected into the tail vein of nude mice to establish a xenograft tumour model. The fluorescence intensity and position of MDA-MB-231-luc cells were used to assess cell metastasis (Fig. 3A, B), while the corresponding lung sections treated with or without AC were analysed using H&E staining (Fig. 3C, D). In the xenograft tumor model by injecting BT549 cells, the cell metastasis was examined by calculating the number of pulmonary metastatic nodules (Fig. 3E, S3A). As previously stated, we found that AC treatment reduced the lung metastasis in MDA-MB-231-luc and BT549 induced nude mice. Similarly, the expression levels of MMP2, MMP3, N-Cadherin, and vimentin were significantly decreased, while the level of E-Cadherin was increased in the lung tissues (Fig. 3F, S3B-F). In addition, immunohistochemistry analysis revealed that MMP2 level decreased, while that of E-Cadherin increased in the MDA-MB-231-luc induced lungs in response to AC treatment (Fig. 3G, H), indicating that AC inhibited the metastasis of BT549 and MDA-MB-231 cells *in vivo* and *in vitro*. Overall, our results demonstrated that AC suppressed the metastasis of human TNBC.

### Anomanolide C induces autophagy-related cell death in TNBC cells

For further reveal the mechanism of AC, the manners of cell death were analyzed. The cell apoptosis inhibitor Z-VAD-FMK (Z-VAD), necrosis inhibitor necrostatin-1 (Nec-1), cell pyroptosis inhibitor disulfiram, cuproptosis inhibitor ammonium tetrathiomolybdate (VI), cell autophagy inhibitor 3-methyladenine (3-MA), and ferroptosis inhibitors Ferrostatin-1 (Fer-1) were used in the experiments. The percentage of cell viability was examined by CCK-8 assay, and the data show that AC was inhibited by Z-VAD after the co-treatment, demonstrating that AC could induce cell apoptosis partially (Fig. S4A). Besides, no significant effect after the combined treatment of AC and Nec-1 was observed, showing that AC could not induce cell necrosis (Fig. S4B). Meanwhile, we also examined the cell viability after treatment with AC and disulfiram or VI simultaneously, and the results indicated that AC could not induce cell pyroptosis and cuproptosis (Fig. S4C, D).

We next verified whether the effect of AC was related to ferroptosis. The Ferrostatin-1 was combined with AC in MDA-MB-231 cells. The effect of AC was inhibited by Fer-1, which lead us to speculate that the mechanism of AC may be achieved by inducing cell ferroptosis (Fig. S4E). As cell ferroptosis inhibitor could not absolutely inhibit the effect of AC, we further examined whether AC could induce autophagy, since withanolides have been reported to activate the autophagy process 48, 49. The effect of AC was inhibited by cell autophagy inhibitor 3-MA, indicating that the effect of AC was achieved partially by inducing cell autophagy (Fig. S4F). To further verify whether the effect of AC could be totally inhibited, we examined the cell viability after the co-treatment with AC, 3-MA and Fer-1, and the result indicated that the effect of AC was inhibited totally after both 3-MA and Fer-1 treatment (Fig. S4G). These results demonstrate that the effect of AC may be achieved by inducing ferroptosis and autophagy of TNBC cells.

**Figure 4:**
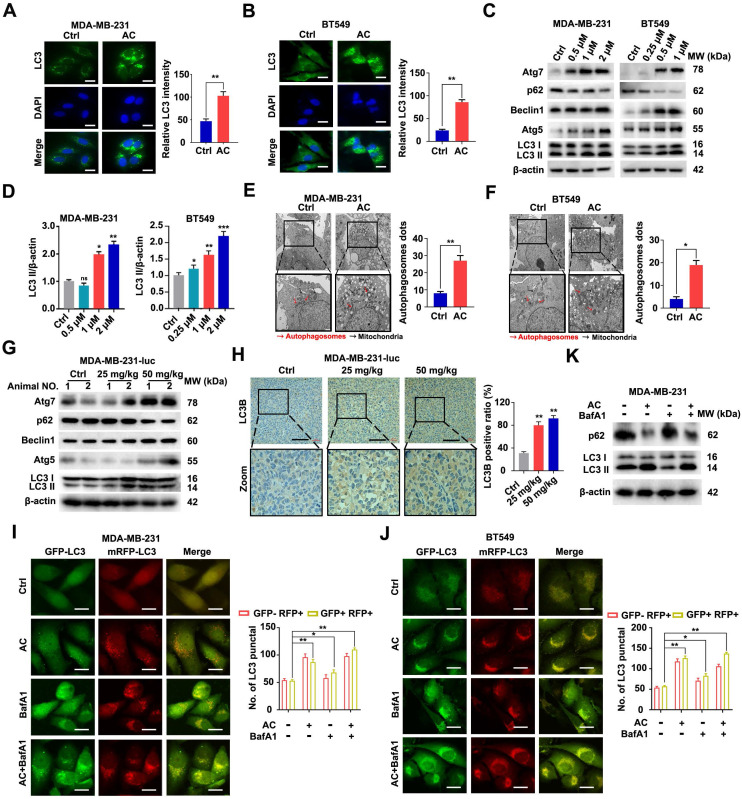
Anomanolide C induces autophagy in TNBC cells *in vitro* and *in vivo*. (A-B) Endogenous LC3B puncta were examined using immunofluorescence in BT549 and MDA-MB-231 cells that were exposed to AC for 24 hours. LC3 quantitation and representative images are shown. Scale bar, 20 µm. (C) MDA-MB-231 and BT549 cells treated with AC at the specified concentration for 24 h were collected and lysed, and the expression of p62, ATG5, ATG7, Beclin1 and LC3 was analyzed using immunoblotting. beta-actin was used as a loading control. (D) Quantification analysis and representative images of LC3 II are shown. (E-F) MDA-MB-231 and BT549 cells were treated with AC in the indicated concentrations for 24h, cells were then observed by transmission electron microscopy. Representative images and quantitative analysis of autophagosome were displayed. (G) Western blotting analysis of MDA-MB-231 xenograft tumor tissues from control or AC (25 and 50 mg/kg) treated nake mice for expression of p62, ATG5, ATG7, Beclin1 and LC3. β-actin was evaluated as a loading control. (H) Immunohistochemical results of LC3B expression in representative MDA-MB-231-luc cells tumor sections of vehicle and AC (25 and 50 mg/kg) treated naked mice. The quantitative analysis of LC3B expression were displayed. (I) immunoblotting analysis the expression of p62, Beclin1 and LC3 in MDA-MB-231 cells treated with or without AC and BafA1. (J-K) After co-incubation with BafA1 (10 nM), BT549 and MDA-MB-231 cells were transfected with GFP/mRFP-LC3 plasmid. Representative images and quantification analysis of LC3 are shown. Scale bar, 20 μm. Data are presented as the mean ± SEM. These results are consistent with those of at least three different experiments. ns, not significant, *, *P* < 0.05, **, *P* < 0.01, ***, *P* < 0.001. Statistical significance was determined relative to the appropriate control groups.

In an effort to further explore the effect of AC on cell autophagy, we examined the occurrence of autophagy after AC treatment. we assessed LC3 in the cells and found that the immunofluorescence intensity of LC3 was significantly increased after AC treatment by using immunofluorescence *in vitro* (Fig. 4A, B). Next, immunoblotting analysis showed that the change of LC3-I to LC3-II was enhanced after treated with AC, the expression of Atg5, Atg7 and Beclin1 also increased, whereas the expression of p62 was suppressed in BT549 and MDA-MB-231 cells (Fig. 4C, D, S5A-H). The cells were observed using a transmission electron microscope (TEM), we found that autophagosomes increased in AC-treated groups as compared with control groups after AC treatment (Fig. 4E, F). Further, we observed that the expression of Atg5, Atg7 and Beclin1 significantly increased in MDA-MB-231-luc induced nude mice model. However, the expression of p62 decreased after treatment with AC *in vivo* (Fig. 4G, S6A-E). The results of immunohistochemical also confirmed this view (Fig. 4H). Meanwhile, we elevated levels of LC3-II may be linked with enhanced autophagosome turnover or autophagosome synthesis. Thus, to further explore the role of AC in autophagy, the RFP-mGFP-LC3 expression vector was also examined to study investigate the fluorescent autophagosomes (yellow) and autolysosomes (red) in MDA-MB-231 and BT549 cells. The autophagy inhibitor, Bafilomycin A1 (BafA1), which could block the fusion between autophagy and lysosome, was co-treatment with AC. The numbers of both of these fluorescent entities increased after treated with AC. The co-treatment of BafA1 and AC leads to the further accumulation of autophagosomes, which indicates that AC-induced autophagy is a continuous process in MDA-MB-231 and BT549 cells (Fig. 4I, J). Besieds, the expression of p62 and LC3 were also examined using western blotting, we found that the accumulation of p62 increased, but LC3 is no significant change upon BafA1 treatment, compared with AC-treated groups in MDA-MB-231 cells, indicating that AC may induce autophagic flux in TNBC cells (Fig. 4K, S7A, B). These outcomes sustain support the view that AC may activate the complete autophagic flux in TNBC cells.

**Figure 5:**
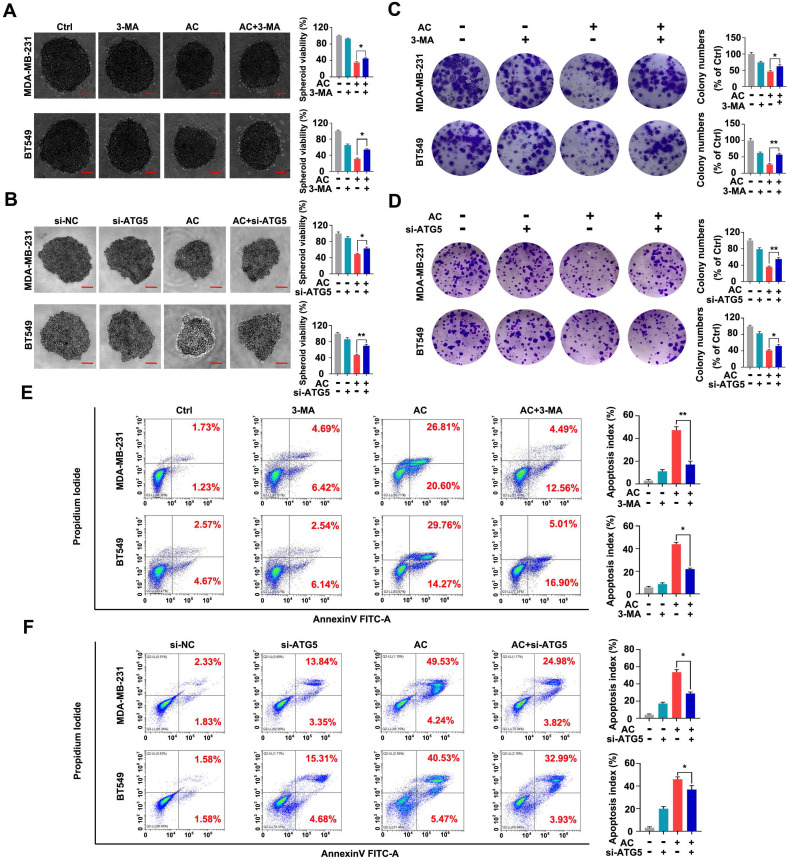
Anomanolide C elicits autophagic cell death in human TNBC cells *in vitro*. (A) MDA-MB-231 and BT549 cells were treated with 3-MA (1mM), respectively. Representative images and quantification of the 3D spheroids viability are presented after treatment with or without AC (MDA-MB-231: 1μM; BT549: 0.5 μM). (B) MDA-MB-231 and BT549 cells were transfected with negative-control or ATG5 siRNA, respectively. Representative images and quantification of the 3D spheroids viability are shown after treatment with or without AC (MDA-MB-231: 1μM; BT549: 0.5 μM). (C) Colony formation of MDA-MB-231 and BT549 cells were shown in the co-treated with or without AC group. (C) Colony formation of MDA-MB-231 and BT549 cells were shown in the co-treated with or without AC group. Quantification of colonies and representative images are shown. (D) MDA-MB-231 and BT549 cells were transfected with negative-control or ATG5 siRNA, respectively. Representative images and quantification of the colonies are shown after treatment with or without AC (MDA-MB-231: 1μM; BT549: 0.5 μM). (E) MDA-MB-231 and BT549 cells were co-treated with or without AC for 24 h, apoptosis ratios were determined by flow cytometry analysis of Annexin-V/PI double staining. Representative images and quantification of apoptosis were shown. (F) MDA-MB-231 and BT549 cells were transfected with negative-control or ATG5 siRNA, respectively. apoptosis ratios were determined by flow cytometry analysis after treatment with or without AC. Representative images and quantification of apoptosis were shown. Data are presented as the mean ± SEM. These results are consistent with those of at least three different experiments. ns, not significant, *, *P* < 0.05, **, *P* < 0.01, ***, *P* < 0.001. Statistical significance was determined relative to the appropriate AC groups.

Autophagy acts as a “double-edged sword” in the development of cancer 50, 51. We further test the relation between AC-induced autophagy and the multiplication inhibition of TNBC cells. The 3-MA or ATG5 silencing was used to block autophagy activation. We found that the viability of 3D spheroids in both BT549 and MDA-MB-231 cells by 3-MA treatment or ATG5 knockdown significantly were higher than those AC-treated groups. Meanwhile, the number of surviving 3D spheroids was enhanced by the co-treated groups, compared with the AC groups (Fig. 5A, B). Similarly, we found that inhibiting autophagy *in vitro* was able to increase colony numbers by colony formation assay (Fig. 5C, D), reaffirming that the multiplication inhibition of TNBC cells owing to AC-treatment was autophagy-dependent. Then, we examine the apoptosis index in BT549 and MDA-MB-231 cells that co-treated with AC and 3-MA or ATG5 knockdown by using flow cytometry analysis. The results confirmed that 3-MA or ATG5 knockdown could partly reverse the cell death induced by AC (Fig. 5E, F). Taken together, these data indicate that AC can induce autophagy-related cell death in TNBC cells.

### Anomanolide C induces ferroptosis in TNBC cells

**Figure 6:**
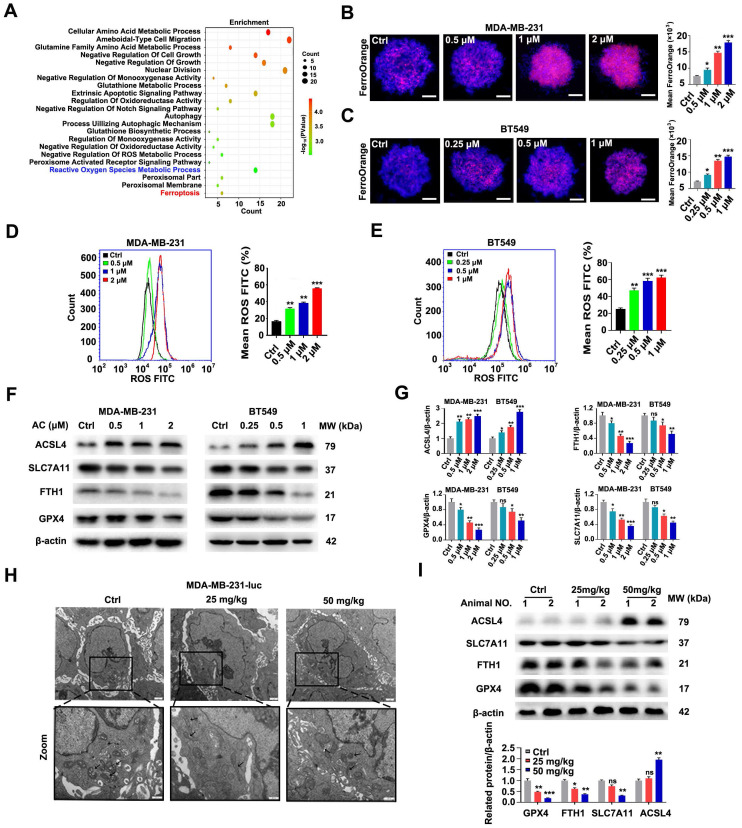
Anomanolide C induces ferroptosis in TNBC cells *in vitro* and* in vivo*. (A) KEGG were performed to predict the potential targets. Functional clustering of gene ontology (GO) was carried out on molecular function. (B-C) Immunofluorescence analysis of Fe^2+^ levels in MDA-MB-231 and BT549 3D spheroids treated with or without AC for 24h. Quantification of immunofluorescence analysis were shown. Scale bar, 20 μm. (D-E) ROS formation in the absence or presence of AC (24 h) was observed by flow cytometry. Quantification of ROS levels and representative images are shown. (F-G) Western blotting analysis expression of FTH1, GPX4, SLC7A11 and ACSL4 treated with or without AC in MDA-MB-231 and BT549 cells. β-actin was used as a loading control. Quantification of FTH1, GPX4, SLC7A11 and ACSL4 levels and representative images are shown. (H) MDA-MB-231-luc treated nake mice tumors were observed by transmission electron microscopy. The arrow represents mitochondria. (I) Immunoblotting analysis expression of FTH1, GPX4, SLC7A11 and ACSL4 treated with AC (25, 50 mg/kg) in nake mice tumor. β-actin was used as a loading control. Quantification of FTH1, GPX4, SLC7A11 and ACSL4 levels and representative images are shown. Data are presented as the mean ± SEM. These results are consistent with those of at least three different experiments. ns, not significant, *, *P* < 0.05, **, *P* < 0.01, ***, *P* < 0.001. Statistical significance was determined relative to the appropriate AC groups.

The obvious mitochondrial shrinkage has been discovered as a significant phenomenon in TNBC cells 52. We then researched the effect of AC inducing cell ferroptosis. Transcriptome data from naked mouse tumors were first analyzed using KEGG, and the findings indicated that the biological functions of AC were mainly involved in ferroptosis, reactive oxygen species metabolic processes, and others (Fig. 6A). We further explored the levels of Fe^2+^ ion in TNBC cells, and discovered that the Fe^2+^ ion levels in the TNBC cells were higher in the AC-treatment group than that of the control group (Fig. 6B, C). The accumulation of intracellular ROS was also increased in TNBC cells after the treatment of AC using flow cytometry (Fig. 6D, E). The mitochondrial ROS of AC-treated 3D spheroids was examined using immunofluorescence and flow cytometry, and the increase of mean fluorescence suggested that AC treatment can lead to mito-ROS accumulation in MDA-MB-231 and BT549 cells (Fig. S8A-D). Mitochondrial membrane potential (MMP) was also suppressed with AC treatment, indicating that AC-induced death triggers the accumulation of ROS in the mitochondria in MDA-MB-231 cells (Fig. S8E, F). Furthermore, the expression of the ferroptosis-associated proteins FTH1, GPX4 and SLC7A11 decreased, while the expression of ACSL4 increased in BT549 and MDA-MB-231 cells after AC-treatment (Fig. 6F, G). The expression of GPX4 was found to reduce after AC treatment by immunofluorescence assay (Fig. S9A). Then, the mitochondrial morphological changes were verified in naked mice tumor induced by MDA-MB-231-luc cells *via* TEM. And the membrane density increased while the mitochondria and cristae shrank after AC treatment, compared with the control group (Fig. 6H). Moreover, we also assayed the expression of key proteins associated with ferroptosis by western blotting *in vivo* (Fig. 6I). Besides, the expression of GPX4 was also decreased *in vivo* by immunohistochemical assay (Fig. S9B). Taken together, these above results demonstrated that AC could induce ferroptosis of TNBC cells.

### Ferroptosis induced by Anomanolide C is related to autophagy activation in TNBC cells

**Figure 7:**
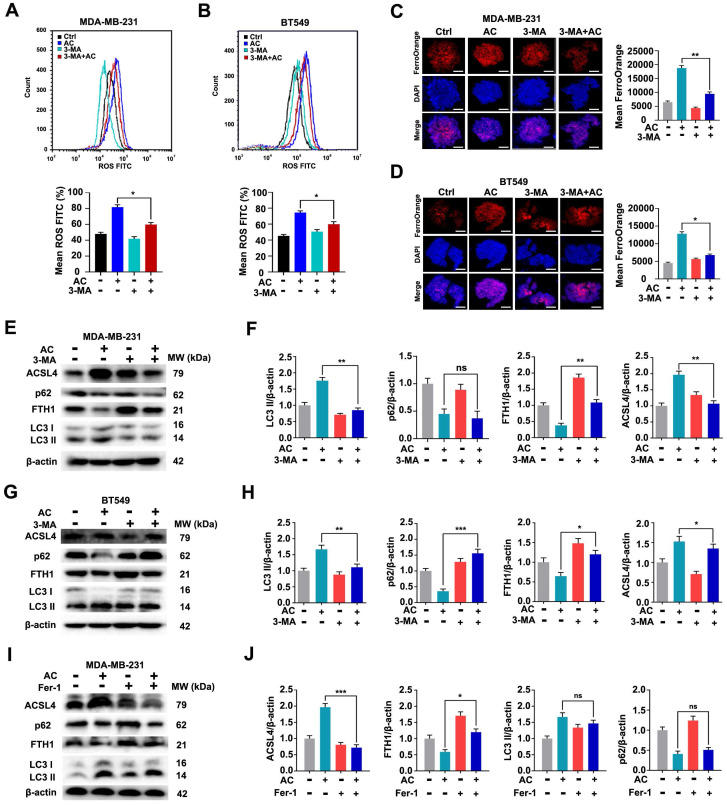
Anomanolide C induces ferroptosis is related to autophagy activation in TNBC cells. (A-B) ROS formation in the absence or presence of AC combine with autophagy inhibitor, 3-MA (1 mM) was observed by flow cytometry. Quantification of ROS levels and representative images are shown. (C-D) Immunofluorescence analysis of Fe^2+^ levels in MDA-MB-231 and BT549 3D spheroids treated with or without AC and 3-MA for 24 h. Quantification of immunofluorescence analysis were shown. Scale bar, 20 μm. (E, G) MDA-MB-231 and BT549 cells co-treated with or without AC and 3-MA for 24 h were collected and lysed, and the expression of p62, LC3, FTH1 and ACSL4 was analyzed using immunoblotting. beta-actin was used as a loading control. (F, H) Quantification of immunoblotting analysis were shown. (I) BT549 cells co-treated with or without AC and ferroptosis inhibitor, Fer-1 for 24 h were collected and lysed, and the expression of p62, LC3, FTH1 and ACSL4 was analyzed using immunoblotting. beta-actin was used as a loading control. (J) Quantification of immunoblotting analysis were shown. Data are presented as the mean ± SEM. These results are consistent with those of at least three different experiments. ns, not significant, *, *P* < 0.05, **, *P* < 0.01, ***, *P* < 0.001. Statistical significance was determined relative to the appropriate AC groups.

It is well-known that ferroptosis is an autophagic cell death process 53. To verify the potential association between the AC-induced ferroptosis and autophagy, we examined the occurrence of ferroptosis after AC treatment. We used an autophagy inhibitor, 3-MA, to block autophagy firstly, and then verified the changes of ferroptosis in MDA-MB-231 and BT549 cells. The levels of ROS in AC-treated TNBC cells were examined by flow cytometry assay (Fig. 7A, B). The results showed that the levels of ROS was decreased after the co-treatment of AC and 3-MA, compared with the AC-treated group, indicating that the activation of autophagy was blocked and the ferroptosis was aslo repressed in TNBC cells. The level of Fe^2+^ ion in TNBC 3D spheroids was then explored,showing that the Fe^2+^ ion level was higher in the AC-treatment group than in the co-treatment group of AC and 3-MA (Fig. 7C, D). We further observed the expression of autophagy proteins LC3 and p62, and ferroptosis proteins FTH1 and ACSL4 by immunoblotting analysis (Fig. 7E-H). The results showed that the expression of FTH1 was enhanced after co-treated with AC and 3-MA, compared with the AC group, whereas the expression of ACSL4 was suppressed in BT549 and MDA-MB-231 cells, indicating that the ferroptosis was inhibited while the activation of autophagy was obstructed in TNBC cells. To further verify whether autophagy was also inhibited while ferroptosis was blocked, we selected a ferroptosis inhibitor, Fer-1, to inhibit ferroptosis. The expression of autophagy proteins LC3 and p62 was examined by western blotting (Fig. 7I, J), and the results demonstrated that autophagy was not inhibited significantly while ferroptosis was inhibited by Fer-1. Taken together, ferroptosis induced by AC is related to autophagy activation in TNBC cells.

### Anomanolide C exerts anti-migration effect *via* autophagy-dependent ferroptosis of TNBC cells

**Figure 8:**
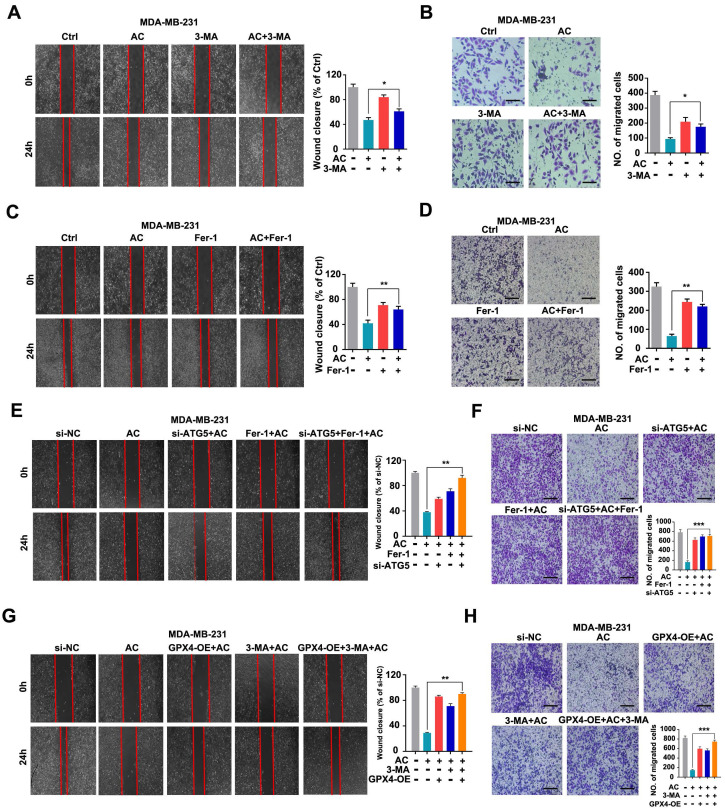
Anomanolide C inhibits TNBC cell migration *in vitro* by causing autophagy-dependent ferroptosis. (A-B) MDA-MB-231 cells were treated for 24 hours with AC (1 µM) alone or in conjunction with 3-MA (1 mM). 3-MA was introduced 6 hours prior to the AC treatment. The ability of the cells to migrate was subsequently evaluated utilizing the transwell assay and scratch test. Representative images and date are shown. Scale bar, 100 µm. (C-D) MDA-MB-231 cells were treated for 24 hours with AC (1 µM) alone or in conjunction with Fer-1 (1 µM). Fer-1 was added 6 hours before AC treatment. The ability of the cells to migrate was then evaluated using the transwell assay and scratch test. Representative images and date are shown. Scale bar, 100 µm. (E-F) MDA-MB-231 cells were transfected with negative-control or ATG5 siRNA. The ability of the cells to migrate was evaluated using the scratch assay and transwell assay after co-treatment with or without AC and Fer-1. Representative images and date are shown. Scale bar, 100 µm. (G-H) MDA-MB-231 cells were transfected with negative-control or overexpression of GPX4. The ability of the cells to migrate was evaluated using the scratch assay and transwell assay after co-treatment with or without AC and 3-MA. Representative images and date are shown. Scale bar, 100 µm. Data are expressed as the mean ± SEM. These results are consistent with those of at least three different experiments. ns, not significant, *, *P* < 0.05, **, *P* < 0.01, ***, *P* < 0.001. Statistical significance was compared between the respective AC groups.

To elucidate the mechanisms by which AC inhibits TNBC cell migration. We evaluated the relationship between autophagy-dependent ferroptosis and migration in AC-treated BT549 and MDA-MB-231 cells. We primarily used AC treated with 3-MA by using scratch and transwell assay and discovered that suppressing autophagy *in vitro* increased the wound healing ratio and quantity of migration in MDA-MB-231 cells, compared with AC group (Fig. 8A, B). Similarly, the results were observed in BT549 cells (Fig. S10A, B). We next combined AC with the Fer-1 and found that the wound healing ratio (Fig. 8C) and quantity of migration (Fig. 8D) were both increased, compared with AC group when ferroptosis was inhibited in MDA-MB-231 cells. Meanwhile, similar results were obtained in BT549 cells (Fig. S10C, D). Thus, AC exerts an anti-migration effect in TNBC cells *via* autophagy and ferroptosis. To further validate the anti-migration effect *via* autophagy-dependent ferroptosis of TNBC cells, the 3-MA or ATG5 silencing was used to block autophagy activation, and Fer-1 or overexpression of GPX4 was used to inhibit ferroptosis. The wound healing ratio and quantity of migration were increased in AC, Fer-1 and ATG5 knockdown co-treatment groups, compared with AC group (Fig. 8E, F). Similarly, the wound healing ratio and quantity of migration were also raised in GPX4 overexpression, 3-MA, and AC co-treatment groups, compared with AC group (Fig. 8G, H). These above results demonstrated that the anti-migration effect of AC was suppressed when autophagy and ferroptosis was inhibited. In summary, AC exerts anti-migration effect *via* autophagy-dependent ferroptosis of TNBC cells.

### Anomanolide C reduces GPX4 protein level *via* directly inducing GPX4 ubiquitination

**Figure 9:**
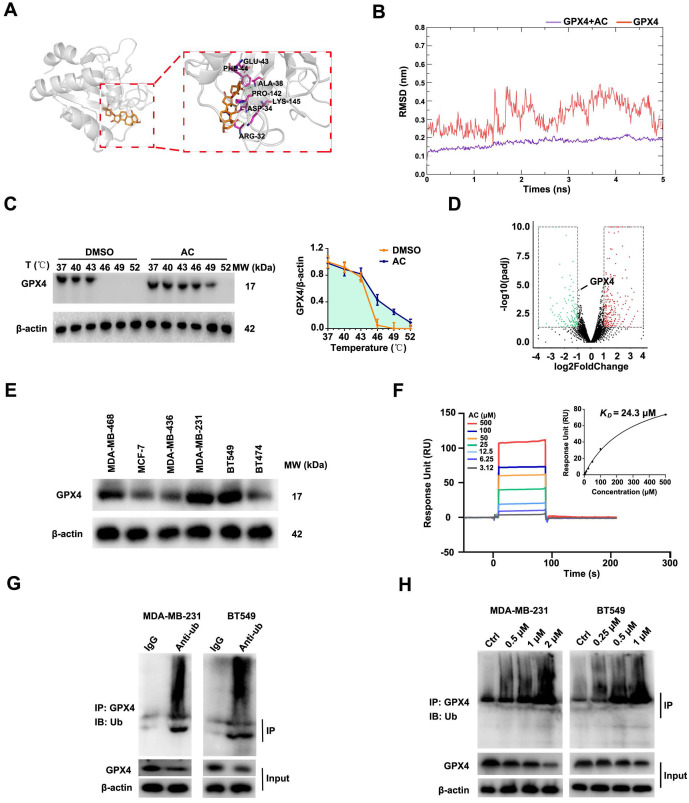
Anomanolide C reduces GPX4 protein level via directly inducing GPX4 ubiquitination. (A) The predicted binding mode of AC with GPX4. (B) The combination of AC and GPX4 by molecular dynamic simulation. (C) CETSA analysis of GPX4 combined with AC. β-actin was measured as a loading control. Images and quantitation of the percentage of positive ratios are represented. (D) Volcano plot of autophagy-related ferroptosis genes with significant differences expression in MDA-MB-231 cells treated with or without AC (1 µM). (E) Immunoblotting analysis GPX4 expression in the breast cancer cell lines. (F) AC binds to GPX4 protein as shown by surface plasmon resonance (SPR) measurements. The estimated *K_D_* is ~24 µM. (G) MDA-MB-231 and BT549 cell lysates were incubated with IgG or anti-Ub antibody at 4 °C for 6 h, then agarose A + G was added, co-incubated at 4 °C overnight on rotary shaker. The samples were washed with PBS and analyzed by immunoblotting assay. (H) AC was added into MDA-MB-231 and BT549 cells and incubated for 24 h. The proteins were collected and incubated GPX4 antibody for 2 h at 4 °C, then agarose G was added and co-incubated at 4 °C overnight. The ubiquitination level of GPX4 was analyzed by immunoblotting assay with anti-Ub antibody. Data are expressed as the mean ± SEM. These results are consistent with those of at least three different experiments. ns, not significant, *, *P* < 0.05, **, *P* < 0.01, ***, *P* < 0.001. Statistical significance was compared between the respective AC groups.

As is known to all that GPX4 was a prominent negative regulator of ferroptosis 54, 55. We predicted the potential targets for AC by Swiss Target Prediction datebases, and found that AC may target GPX4. AC was found to form a hydrophobic interaction with the indole side chain of ARG-32, PRO-142, and PHE-44, as well as hydrogen bonds with ALA-38, LYS-145 and ASP-34 (Fig. 9A). Moreover, the AC/GPX4 complex exhibited conformational curves after 5 ns simulation, indicating that GPX4 is a key protein for binding with AC through molecular dynamics (Fig. 9B). To further verified the target of AC, we validated the binding capability of AC to GPX4 through Cellular Thermal Shift Assay (CETSA), suggesting that AC could bind to GPX4 and enhance the stability of GPX4 (Fig. 9C). We then validated the changes of GPX4 gene *via* RNA-seq analysis, and the results showed that the gene of GPX4 did not change obviously after AC treated MDA-MB-231-luc cells (Fig. 9D). In addition, we also validated the expression of GPX4 at mRNA levels by qPCR assay (Fig. S11), and the results demonstrated that no notable changes in mRNA levels of GPX4 expression. Hence, we predicted, and further validated, whether AC affected GPX4 changes at protein levels. Immunoblotting results demonstrated that the high-expression of GPX4 protein level in MDA-MB-231 and BT549 cells (Fig. 9E). Meanwhile, AC exhibited significant binding affinity to GPX4 protein with an estimated *K_D_* constant of ~24 μM (Fig. 9F). These results demonstrated that AC could reduce the protein level of GPX4 in TNBC cells.

In the ubiquitination procedure, ubiquitin is covalently bound to targets under the catalysis of a whole string of enzymes 56. Hence, we rationalized that another mode of cell death inducing ferroptosis would be the induction of GPX4 ubiquitination. Furthermore, we validated the ubiquitination of GPX4 after AC treatment by co-immunopre-cipitation (Co-IP) analysis (Fig. 9G), and GPX4 was found to be ubiquitinated in TNBC cells. Moreover, the ubiquitination of GPX4 after the treatment with AC was also analyzed (Fig. 9H). These results indicated that AC could improve the ubiquitination of GPX4 in TNBC cells. In summary, AC could decrease GPX4 expression by inducing ubiquitination of GPX4 in TNBC cells.

### Anomanolide C exerts anti-proliferative and anti-migration effects *via* ubiquitinating GPX4 in TNBC cells

**Figure 10:**
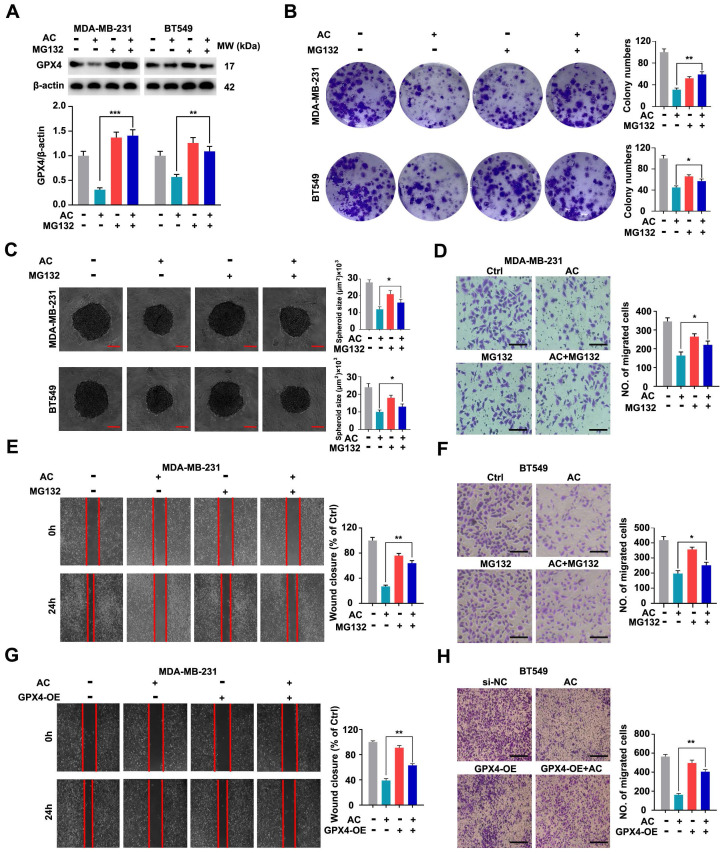
Anomanolide C exerts anti-proliferative and anti-migration effects *via* ubiquitinating GPX4 in TNBC cells. (A) MDA-MB-231 and BT549 cells co-treated with or without AC and MG132 (100 nM) for 24 h were collected and lysed, and the expression of GPX4 was analyzed using immunoblotting. beta-actin was used as a loading control. Quantification of GPX4 levels and representative images are shown. (B) Colony formation of MDA-MB-231 and BT549 cells were tested in the co-treated with or without AC and MG132 group. Quantification of colonies and representative images are shown. (C) MDA-MB-231 and BT549 3D spheroids co-treated with or without AC and MG132 were studied. Quantification of 3D spheroids volume and representative images are shown. (D-E) MDA-MB-231 cells were treated for 24 hours with AC (1 µM) alone or in conjunction with MG132. MG132 was introduced 6 hours prior to the AC treatment. The ability of the cells to migrate was subsequently evaluated utilizing the transwell assay. Representative images and date are shown. Scale bar, 100 µm. (F) BT549 cells were treated for 24 hours with AC (0.5 µM) alone or in conjunction with MG132. MG132 was introduced 6 hours prior to the AC treatment. The ability of the cells to migrate was subsequently evaluated utilizing scratch test. Representative images and date are shown. Scale bar, 100 µm. (G-H) MDA-MB-231 cells were transfected with negative-control or overexpression of GPX4. The ability of the cells to migrate was evaluated using the scratch assay and transwell assay after co-treatment with or without AC. Data are expressed as the mean ± SEM. These results are consistent with those of at least three different experiments. ns, not significant, *, *P* < 0.05, **, *P* < 0.01, ***, *P* < 0.001. Statistical significance was compared between the respective AC groups.

Accumulating evidence has revealed that the ubiquitination of GPX4 could inhibit the growth of cancer cells 57, 58. To investigate whether the ubiquitination of GPX4 inhibits TNBC proliferation and migration, MDA-MB-231 and BT549 cells were firstly treated with or without the proteasome inhibitor MG132 (Fig. 10A). The results demonstrated that GPX4 was accumulated with the treatment of MG132, compared with the AC group, and the degradation of GPX4 is achieved by ubiquitin-proteasome system in MDA-MB-231 cells. Moreover, we discovered that the colony numbers of TNBC cells were significantly higher than AC-treated group (Fig. 10B). Similarly, the volume of 3D spheroids in both MDA-MB-231 and BT549 cells were increased by the co-treated groups, compared with the AC group (Fig. 10C). Besides, we primarily used AC co-treatment with the MG132 to analyze its anti-migration ability by scratch and transwell assay, and the wound closure rate was observed to increase in MDA-MB-231 cells, compared with the AC group (Fig. 10D, E). Meanwhile, similar results were obtained in BT549 cells (Fig. 10F). The results demonstrate that AC exerts anti-migration and anti-proliferative effects on ubiquitinating GPX4 in TNBC cells.

To further demonstrate whether the anti-proliferative and anti-migration effects of AC are related to GPX4, the specifically overexpressed RNAs was transfected into MDA-MB-231 cells to induce GPX4 expression (Fig. S12). We found that the colony numbers of TNBC cells were significantly higher than those in AC-treated group (Fig. S13A). Moreover, the volume of 3D spheroids in both MDA-MB-231 and BT549 cells was also increased by the co-treated groups, compared with the AC group (Fig. S13B). These results demonstrated that AC exerted anti-proliferative effects in GPX4-overexpressed TNBC cells. Furthermore, the wound healing ratio and quantity of migration were increased in TNBC cells after GPX4 overexpression treatment, compared with the AC group (Fig. 10G, H), indicating that GPX4 overexpression caused the anti-migration effects in TNBC cells. Taken together, AC exerts anti-migration and anti-proliferative effects by ubiquitinating GPX4 in TNBC cells.

## Discussion

It is important to clarify the potential targets and relevant mechanisms and of natural products for their further development as new drugs. Anomanolide C (AC) is a major active constituent of *Tubocapsicum anomalum* 38, 59, 60, showing potent anti-cancer activities. However, imprecise mechanisms and targets greatly hinder its development as a candidate for anti-cancer drugs. In this study, we found that AC has significant antitumor activity on TNBC *in vitro* and *in vivo*, including by inhibition of proliferation and blocking of metastasis. We try to explore the potential anti-tumor mechanism of AC from the perspective of regulated cell death (RCD) subroutines. Interestingly, both ferroptosis inhibitor and autophagy inhibitor have shown significant suppressive effects on the antitumor activity of AC, indicating that ferroptosis and autophagy may be critical pathways for AC to treat breast cancer.

In recent years, the anti-tumor mechanism of natural products by inducing autophagy-dependent ferroptosis has been widely reported. For example, chrysin induces autophagy-dependent ferroptosis through targeting CBR1 in pancreatic cancer cells 61, paclitaxel enhances autophagy-dependent ferroptosis pathway by inhibiting glioblastoma 62. These both two reports only demonstrated that natural products can induce autophagy-dependent ferroptosis in cancers. However, the relationship between autophagy and ferroptosis has not been explored in detail. In our study, we further indicated that autophagy was also inhibited while ferroptosis was blocked. Instead, autophagy was not inhibited significantly when ferroptosis was inhibited. This provides new insights into the correlation between autophagy and ferroptosis in natural products.

Indeed, mixed types of cell death appear to be more prevalent in human disease than “pure” types 63, although one type of cell death may dominate over others at a particular stage. In our study, AC mediates TNBC occurrence of a mixed type of cell death based on ferroptosis and autophagy. AC promotes the occurrence of TNBC ferroptosis by inducing autophagy-dependent degradation of GPX4. In addition, autophagy acts as an upstream signal to regulate AC-mediated iron death. AC-induced GPX4 ubiquitination is a central event of crosstalk between autophagy and ferroptosis. This is similar to several selective autophagy that all play an important role in ferroptosis, like ferritinophagy, lipophagy, and clockophagy 64. We report for the first time that a withanolide exerts antitumor activity as an autophagy-dependent ferroptosis inducer, which enriches the anti-tumor mechanism and research direction of withanolides, and provides a reference for the study of autophagy-dependent ferroptosis.

In summary, our results demonstrate that AC can reduce GPX4 protein level by inducing GPX4 ubiquitination and then induces autophagy-dependent ferroptosis in TNBC. Moreover, our findings link GPX4 to cell progression and metastasis *via* ubiquitinating GPX4 in TNBC. Therefore, these findings will shed new light on exploiting AC as a candidate drug from natural origin to improve breast cancer therapy in the future.

## Supplementary Material

Supplementary figures and table.

## Abbreviations

3-MA: 3-Methyladenine
1% PS: 1% penicillin-streptomycin
4% PFA: 4% paraformaldehyde
AC: Anomanolide C
ATCC: American Type Culture Collection
BC: Breast cancer
CETSA: Cellular Thermal Shift Assay
Co-IP: Co-immunoprecipitation
DMEM: Dulbecco's Modified Eagle Medium
FTH1: ferritin H (heart/heavy) chain 1
FTL: ferritin light chain
FBS: Fetal Bovine Serum
Fer-1: Ferrostatin-1
KEGG: Kyoto Encyclopedia of Genes and Genomes
MMP: Mitochondrial membrane potential
mito-ROS: Mitochondrial reactive oxygen species
MD: molecular dynamics
Nec-1: Necrostatin-1
PBS: phosphate buffer saline
RCD: Regulated Cell Death
RPMI: Roswell Park Memorial Institute medium
ROS: Reactive Oxygen Species
SPR: Surface Plasmon Resonance
TCM: Traditional Chinese medicine
TEM: Transmission electron microscope
qRT-PCR: Quantitative real time PCR
