# Supplementary material for: Anomanolide C suppresses tumor progression and metastasis by ubiquitinating GPX4-driven autophagy-dependent ferroptosis in triple negative breast cancer
Source: Int J Biol Sci. 2023 May 8;19(8):2531–50. doi: 10.7150/ijbs.82120 (PMC10197885; doi:10.7150/ijbs.82120)
Supplement: Supplementary file 1 — Supplementary figures and table. [file ijbsv19p2531s1.pdf]

Supplementary materials for

Anomanolide C suppresses tumor progression and metastasis by ubiquitinating GPX4-driven autophagy-dependent ferroptosis in triple negative breast cancer

Yan-Mei Chen[1,a], Wei Xu[3,a], Yang Liu[1,a], Jia-Hui Zhang[1,2], Yuan-Yuan Yang[2], Zhi-wen Wang[2], De-Juan Sun[1, *], Hua Li[1, *], Bo Liu[2, *], Li-Xia Chen[1, *]

1. Wuya College of Innovation, School of Traditional Chinese Materia Medica, Key Laboratory of Structure-Based Drug Design & Discovery, Ministry of Education, Shenyang Pharmaceutical University, Shenyang, 110000, China.

2. State Key Laboratory of Biotherapy and Cancer Center, West China Hospital, Sichuan University, Chengdu, 610041, China.

3. Institute of Structural Pharmacology & TCM Chemical Biology, College of Pharmacy, Fujian University of Traditional Chinese Medicine, Fuzhou 350122, China.

[a]These authors contributed equally to this work.

Corresponding authors: Li-xia Chen, Wuya College of Innovation, School of Traditional Chinese Materia Medica, Key Laboratory of Structure-Based Drug Design & Discovery, Ministry of Education, Shenyang Pharmaceutical University, No. 103, Wenhua Road, Shenyang, 110000, China. E-mail:

syzyclx@163.com; Bo Liu, State Key Laboratory of Biotherapy and Cancer Center, West China Hospital, No. 17, Renmin south Road, Sichuan University, Chengdu, 610041, China. E-mail: liubo2400@163.com; Hua Li, Wuya College of Innovation, School of Traditional Chinese Materia Medica, Key Laboratory of Structure-Based Drug Design & Discovery, Ministry of Education, Shenyang Pharmaceutical University, No. 103, Wenhua Road, Shenyang, 110000, China. E-mail: li_hua@hust.edu.cn; De-juan Sun, Wuya College of Innovation, School of Traditional Chinese Materia Medica, Key Laboratory of Structure-Based Drug Design & Discovery, Ministry of Education, Shenyang Pharmaceutical University, No. 103, Wenhua Road, Shenyang, 110000, China. E-mail: 13693476164@163.com.

This file includes:

Supplementary figures S1 to S13.

Supplementary tables S1.

Supplementary Figure 1.

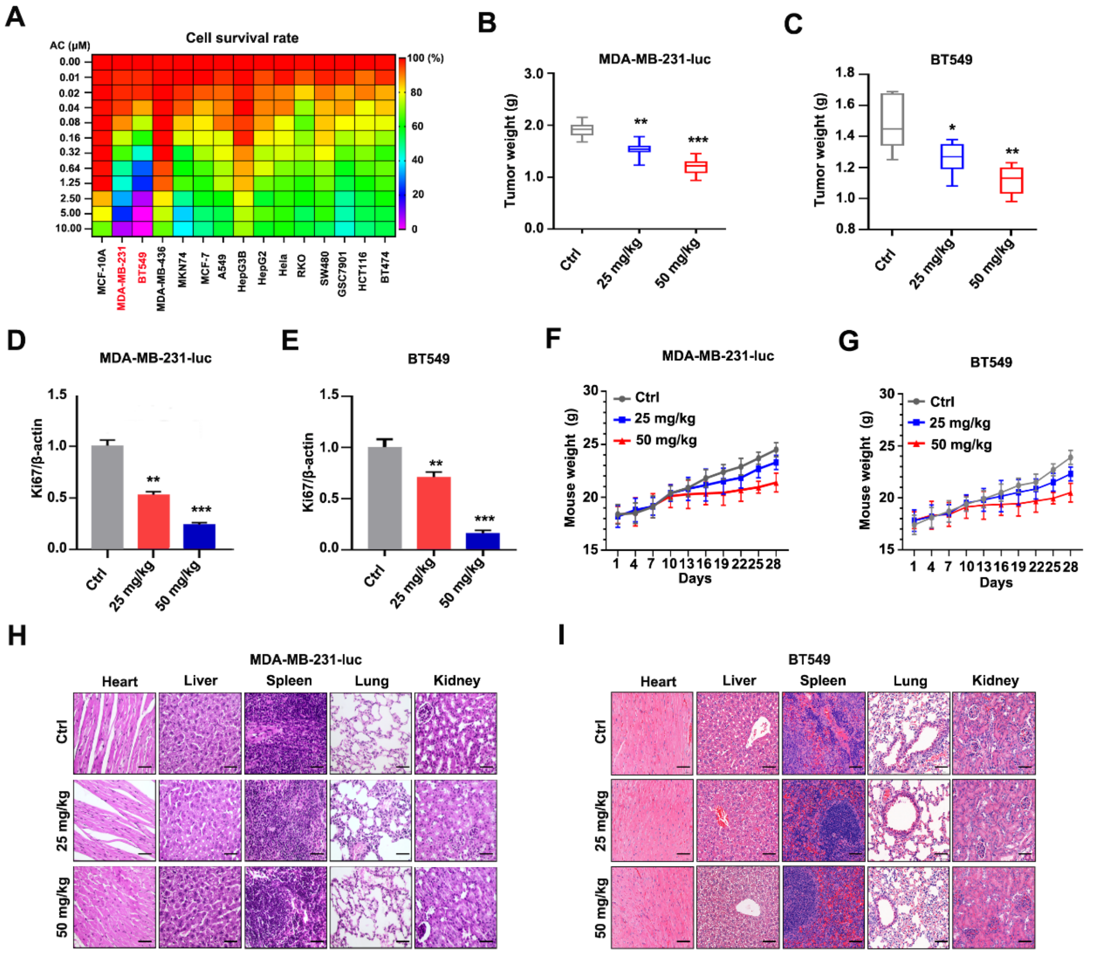

Fig. S1 Anomanolide C inhibits breast cancer cells *in vivo* and *in vitro*. (A) Heat map of mean cell survival values of AC in multiple tumor cell lines at different concentration. Cell viabilities were measured by CCK8 assay, and the cell survival values were calculated by Prism 8.0. (B-C) Quantitative analysis of the MDA-MB-231-luc (B) or BT549 (C) cells tumor weight. (D-E) The statistical diagram of Ki67 protein in MDA-MB-231-luc (D) or BT549 (E) cells tumors. (F-G) Quantitative analysis of the MDA-MB-231-luc (F) or BT549 (G) cells treated mice weight. (H-I) H&E staining of heart, liver, spleen, lung, kidney of the experimental mouse. There has been no obvious toxicity of AC-induced nude mouse. Data are presented as mean ± SEM. Date are from at least three separate experiments. ns, not significant, *, $P < 0.05$, **, $P < 0.01$, ***, $P < 0.001$. Statistical significance was determined relative to the respective control groups.

Supplementary Figure 2.

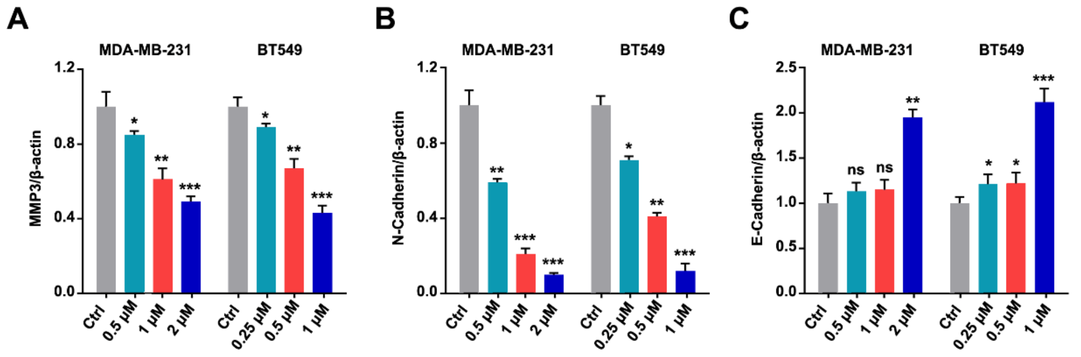

Fig. S2 （A-C）Quantitative analysis of the MMP3, N-Cadherin, E-Cadherin protein in MDA-MB-231 and BT549 cells after AC treatment 24 h. Data are presented as mean ± SEM. Date are from at least three separate experiments. ns, not significant, *, *P* < 0.05, **, *P* < 0.01, ***, *P* < 0.001. Statistical significance was determined relative to the respective control groups.

Supplementary Figure 3.

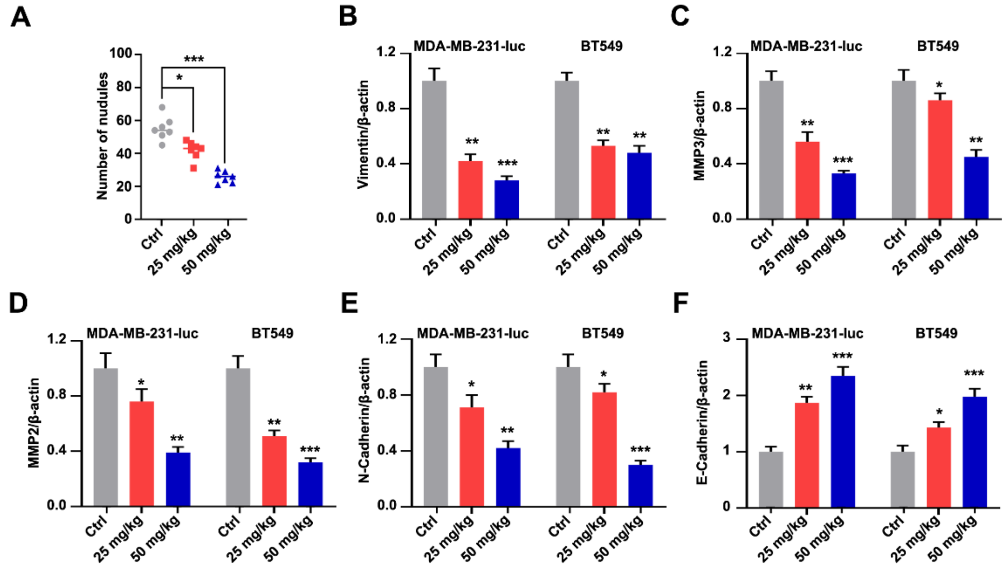

Fig. S3 (A) Tumor cell metastasis was examined by counting metastatic nodules in mouse lung. (B-F) Quantitative analysis of the vimentin, MMP3, MMP2, N-Cadherin, and E-Cadherin protein in MDA-MB-231 and BT549 cells induced nude mice tumor which treated with or without AC. Data are presented as mean ± SEM. Date are from at least three

separate experiments. ns, not significant, *, $P < 0.05$, **, $P < 0.01$, ***, $P < 0.001$. Statistical significance was determined relative to the respective control groups.

Supplementary Figure 4.

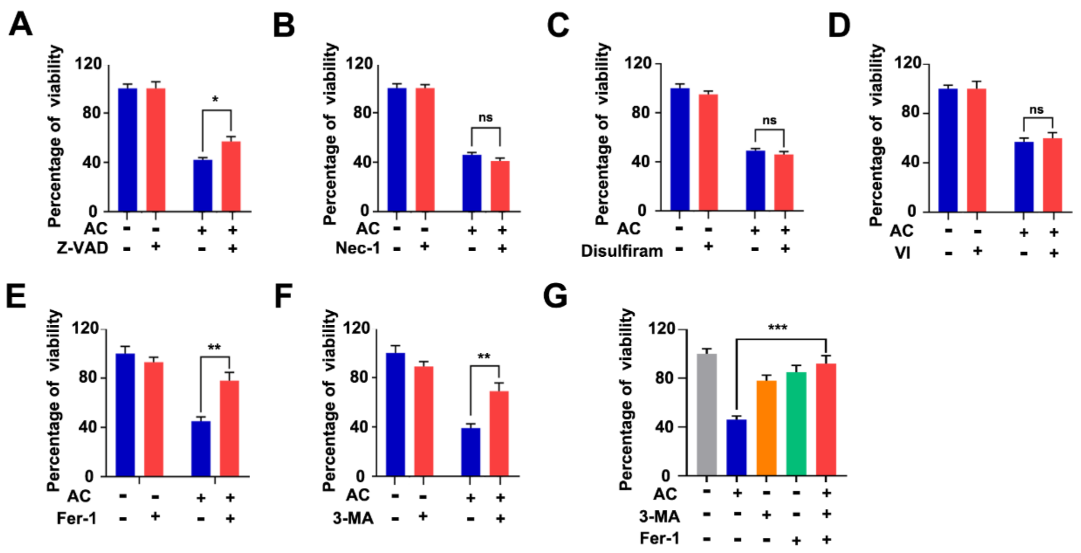

Fig. S4 (A-F) The MDA-MB-231 cell viability of programmed cell death, containing apoptosis (apoptosis inhibitor, Z-VAD, 40 μM), necroptosis (necroptosis inhibitor, Nec-1, 30 μM), pyroptosis (pyroptosis inhibitor, disulfiram, 10 μM), cuproptosis (cuproptosis inhibitor, ammonium tetrathiomolybdate(VI), 5 mg/kg), ferroptosis (ferroptosis inhibitor, Fer-1, 1 μM), autophagy (autophagy inhibitor, 3-MA, 1 mM) were analyzed after co-treatment with AC by CCK8 assay. (G) Quantitative analysis of the MDA-MB-231 cell viability after co-treatment with or without AC, Fer-1, and 3-MA. Data are presented as mean ± SEM. Date are from at least three separate experiments. ns, not significant, *, $P < 0.05$, **, $P < 0.01$, ***, $P < 0.001$. Statistical significance was determined relative to the respective control groups.

Supplementary Figure 5.

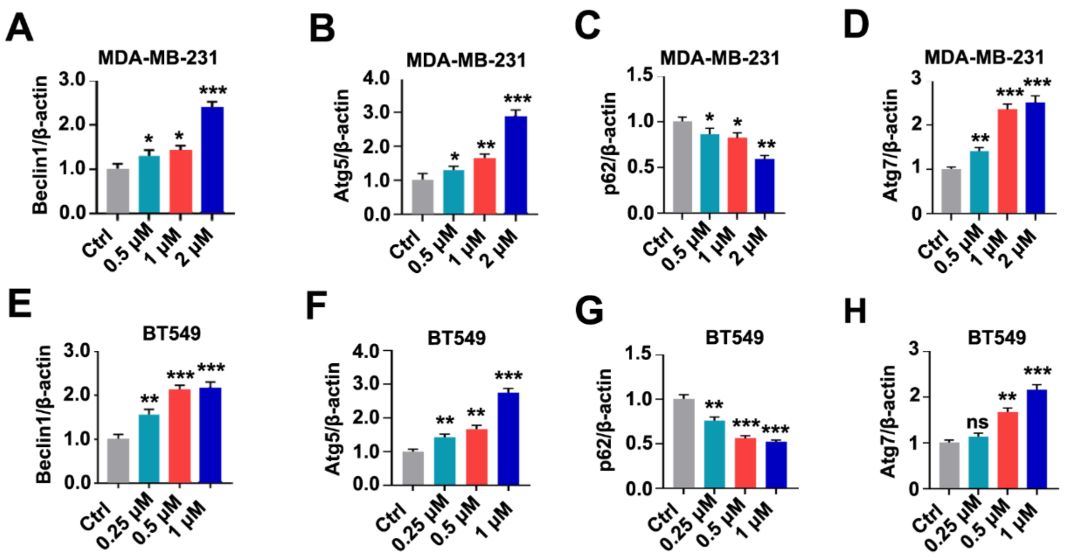

Fig. S5 (A-D) The protein expression of ATG5, ATG7, Beclin1 and p62 in MDA-MB-231 cells which treated with AC. (D-H) The protein expression of ATG5, ATG7, Beclin1 and p62 in BT549 cells which treated with AC. Data are presented as mean ± SEM. Date are from at least three separate experiments. ns, not significant, *, $P < 0.05$, **, $P < 0.01$, ***, $P < 0.001$. Statistical significance was determined relative to the respective control groups.

Supplementary Figure 6.

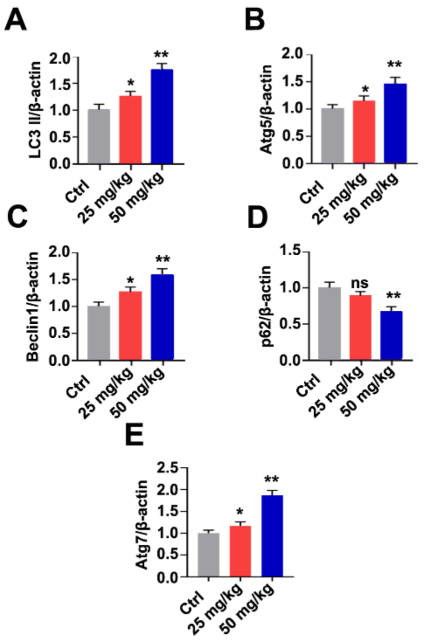

Fig. S6 (A-E) Quantitative analysis of the protein expression of LC3 II, Atg5, Beclin1, p62,

and Atg7 which in MDA-MB-231-luc cells induced nude mice tumor, after AC treatment. Data are presented as mean ± SEM. Date are from at least three separate experiments. ns, not significant, *, P < 0.05, **, P < 0.01, ***, P < 0.001. Statistical significance was determined relative to the respective control groups.

Supplementary Figure 7.

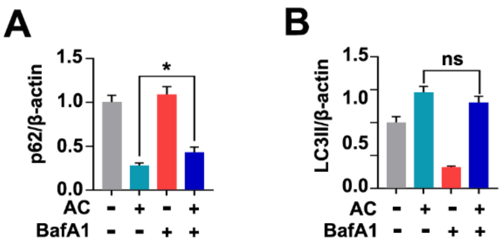

Fig. S7 (A-B) Quantitative analysis of the protein expression of p62, and LC3 II after co-treated with or without AC and BafA1. Data are presented as mean ± SEM. Date are from at least three separate experiments. ns, not significant, *, P < 0.05, **, P < 0.01, ***, P < 0.001. Statistical significance was determined relative to the respective control groups.

Supplementary Figure 8.

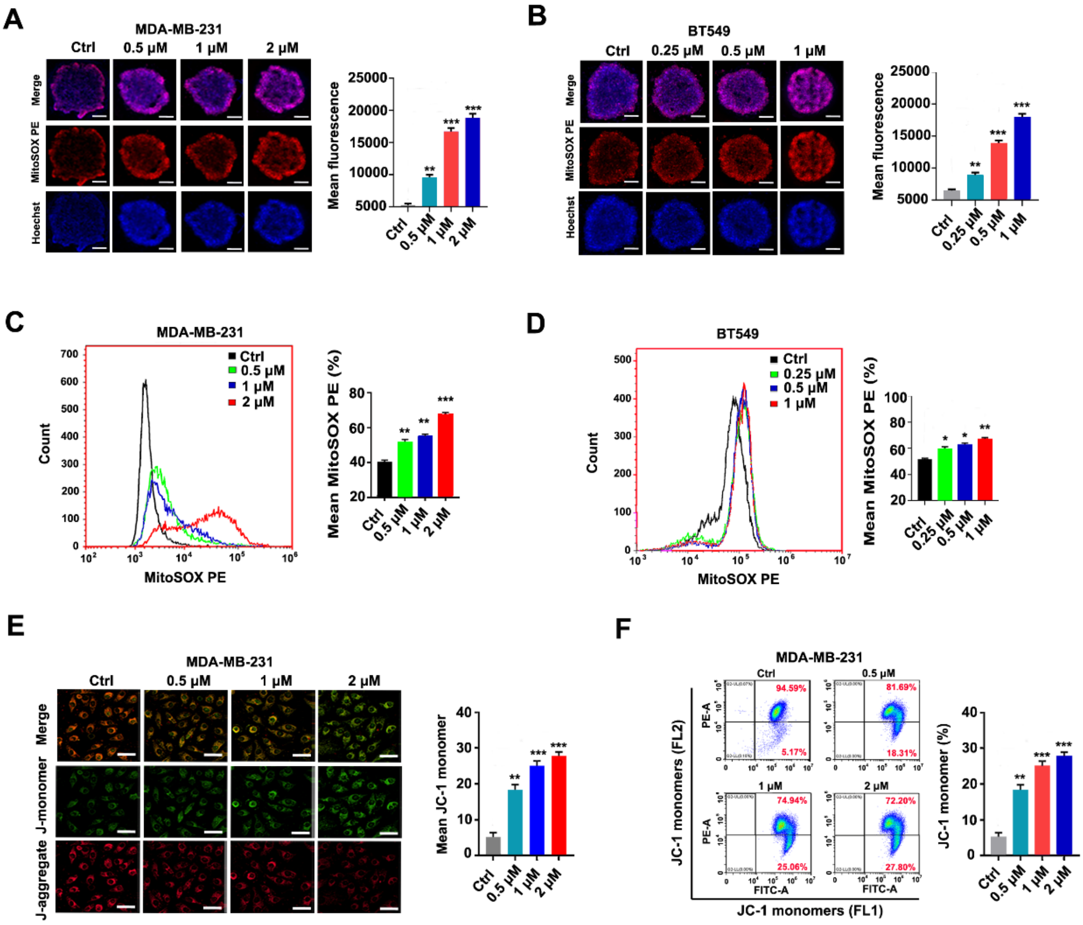

Fig. S8 (A-B) Immunofluorescence analysis of mito-ROS levels in MDA-MB-231 (A) or BT549 (B) 3D spheroids treated with AC for 24 h. Quantification of immunofluorescence analysis were shown. Scale bar, 20 μm. (C-D) The mito-ROS levels of MDA-MB-231 (C) or BT549 (D) cells were detected by flow cytometry with treated AC. Representative images and quantitative analysis of mito-ROS levels were shown. (E) Immunofluorescence analysis of Mitochondrial membrane potential (MMP) levels in MDA-MB-231 cells. Representative images and quantitative analysis of MMP levels were shown. (F) MMP levels in MDA-MB-231 cells were detected by flow cytometry using JC-1. Representative images and quantitative analysis of MMP levels were shown. Data are presented as mean ± SEM. Date are from at least three separate experiments. ns, not significant, *, $P < 0.05$, **, $P < 0.01$, ***, $P < 0.001$. Statistical significance was determined relative to the respective control groups.

Supplementary Figure 9.

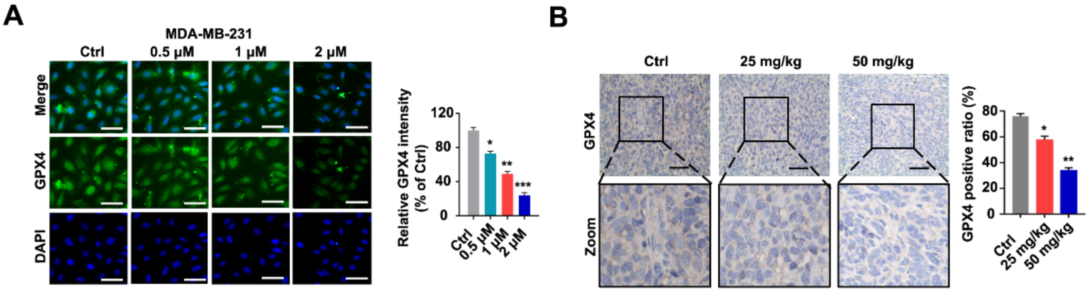

Fig S9. (A) Immunofluorescence analysis of GPX4 levels in MDA-MB-231 cells treated with or without AC for 24 h. Quantitative analysis of GPX4 fluorescence levels were shown. (B) Expression of GPX4 in tumors of naked mice from control and AC (25 and 50 mg/kg)-treated groups. The percentage of positive ratios was quantitatively analyzed and is shown in the images. Scale bar, 40 μm. Data are presented as mean ± SEM. Date are from at least three separate experiments. ns, not significant, *, $P < 0.05$, **, $P < 0.01$, ***, $P < 0.001$. Statistical significance was determined relative to the respective control groups.

Supplementary Figure 10.

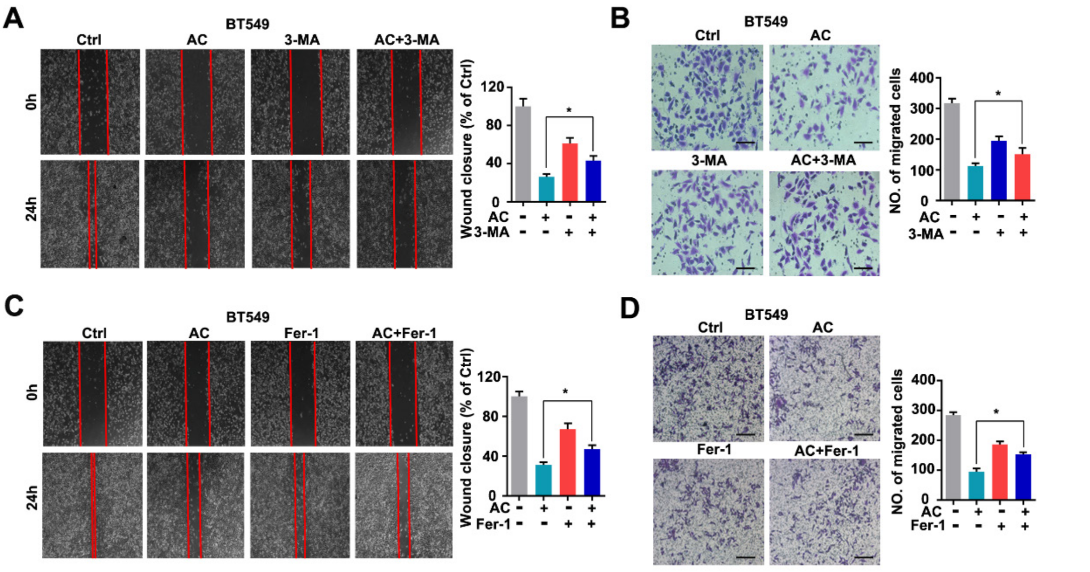

Fig. S10 (A-B) BT549 cells were treated for 24 hours with AC (0.5 μM) alone or in combination with 3-MA (1 mM); 3-MA was administered 6 hours prior to the AC treatment. The cells migration' capacities were then evaluated using the scratch and transwell assays. Representative images and date are shown. Scale bar, 100 μm. (C-D) BT549 cells were

treated for 24 hours with AC (0.5 μM), either alone or in combination with Fer-1 (1 μM), Fer-1 treatment was 6 6 hours prior to the AC. The ability of the cells to migrate was evaluated using the scratch assay and transwell assay. Representative images and date are shown. Scale bar, 100 μm. Data are presented as mean ± SEM. Date are from at least three separate experiments. ns, not significant, *, $P < 0.05$, **, $P < 0.01$, ***, $P < 0.001$. Statistical significance was determined relative to the respective control groups.

Supplementary Figure 11.

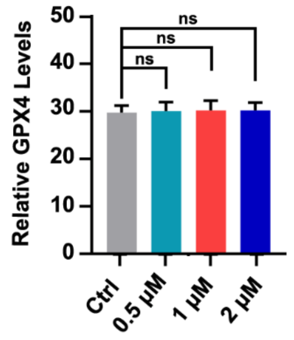

Fig. S11 The expression of GPX4 was tested by qRT-PCR analysis in MDA-MB-231 cells treated with or without AC (1 μM). Data are presented as mean ± SEM. Date are from at least three separate experiments. ns, not significant, *, $P < 0.05$, **, $P < 0.01$, ***, $P < 0.001$. Statistical significance was determined relative to the respective control groups.

Supplementary Figure 12.

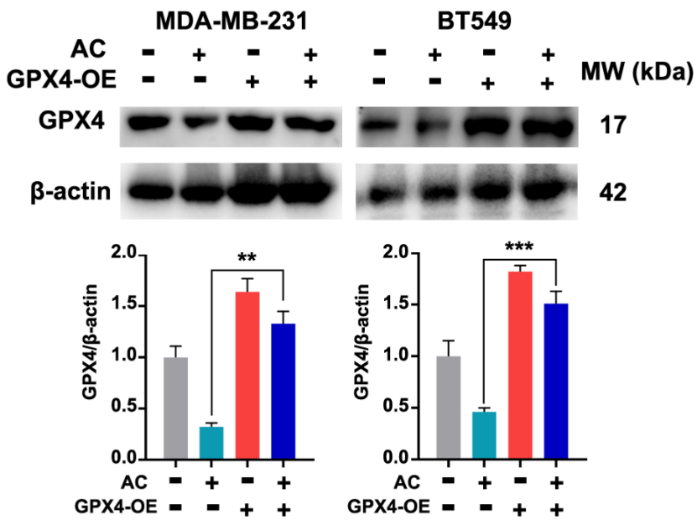

Fig. S12 MDA-MB-231 and BT549 cells were transfected with control or overexpression of GPX4. Besides, the expression levels of GPX4 were determined by immunoblotting analysis. β-Actin was measured as the loading control. Quantification analysis of GPX4 expression is shown. Data are presented as mean ± SEM. Date are from at least three separate experiments. ns, not significant, *, $P < 0.05$, **, $P < 0.01$, ***, $P < 0.001$. Statistical significance was determined relative to the respective control groups.

Supplementary Figure 13.

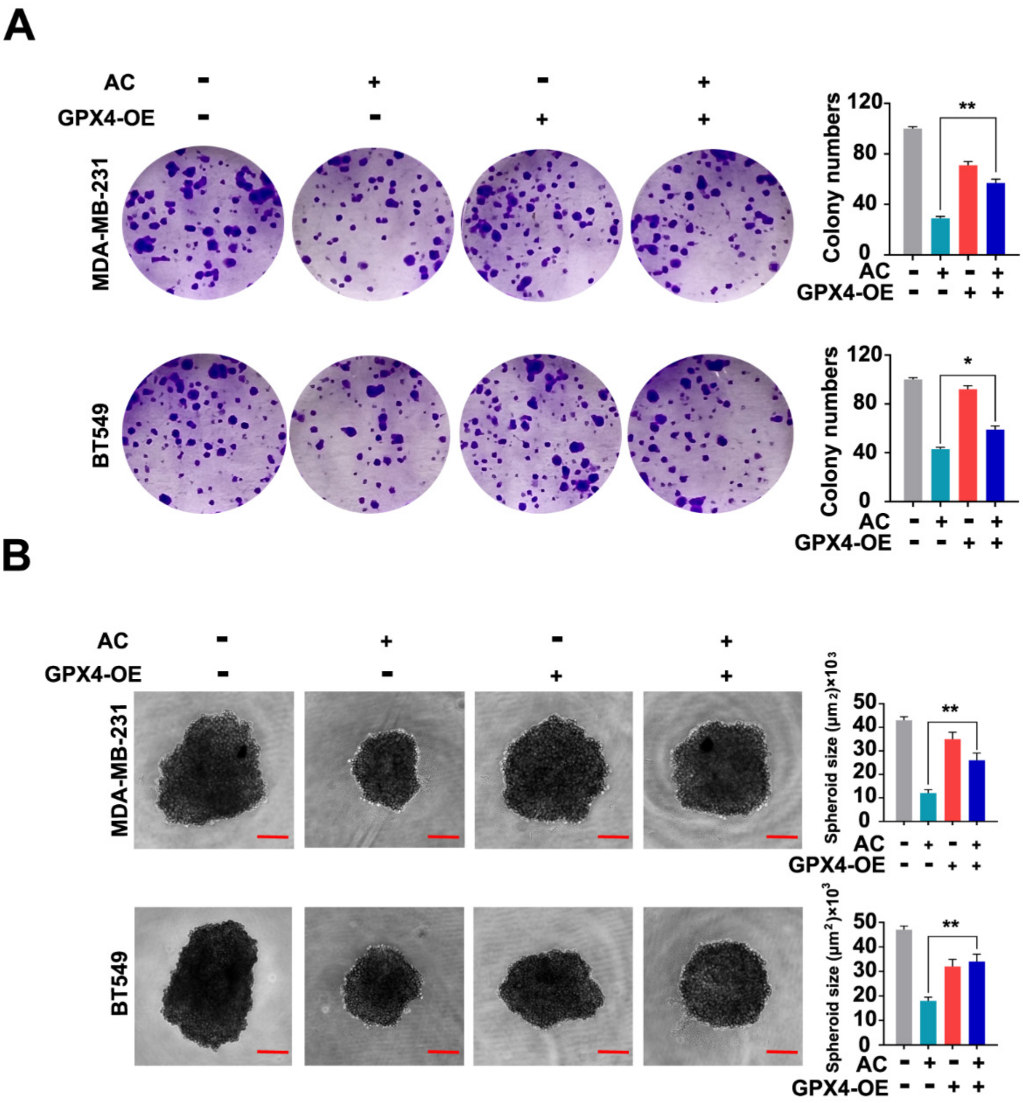

Fig. S13 (A) Colony formation of MDA-MB-231 and BT549 cells were tested in the co-treated with or without AC and overexpression of GPX4 group. Quantification of colonies and representative images are shown. (C) MDA-MB-231 and BT549 3D spheroids co-treated with or without AC and overexpression of GPX4 were studied. Quantification of 3D spheroids volume and representative images are shown. Data are presented as mean ± SEM. Date are from at least three separate experiments. ns, not significant, *, $P < 0.05$, **, $P < 0.01$, ***, $P < 0.001$. Statistical significance was determined relative to the respective control groups.

Table S1. Primer sequences for qRT-PCR

Gene	Forward Primer (5'-3')	Reverse Primer (5'-3')
Actin	CATGTACGTTGCTATCCAGGC	CTCCTTAATGTCACGCACGAT
GPX4	AAGTTCAGTCAGAGACCTGCG	ATATCCGAGCCCTCCTCCTTC
